# Tetraploidy causes chromosomal instability in acentriolar mouse embryos

**DOI:** 10.1038/s41467-019-12772-8

**Published:** 2019-10-23

**Authors:** Lia Mara Gomes Paim, Greg FitzHarris

**Affiliations:** 1 Centre de Recherche du Centre Hospitalier de l’Université de Montréal, H2X 0A9 Montreal, QC Canada; 2 Département d’Obstétrique-Gynécologie, Université de Montréal, H3T 1C5 Montreal, QC Canada

**Keywords:** Chromosome segregation, Kinetochores, Genomic instability

## Abstract

Tetraploidisation is considered a common event in the evolution of chromosomal instability (CIN) in cancer cells. The current model for how tetraploidy drives CIN in mammalian cells is that a doubling of the number of centrioles that accompany the genome doubling event leads to multipolar spindle formation and chromosome segregation errors. By exploiting the unusual scenario of mouse blastomeres, which lack centrioles until the ~64-cell stage, we show that tetraploidy can drive CIN by an entirely distinct mechanism. Tetraploid blastomeres assemble bipolar spindles dictated by microtubule organising centres, and multipolar spindles are rare. Rather, kinetochore-microtubule turnover is altered, leading to microtubule attachment defects and anaphase chromosome segregation errors. The resulting blastomeres become chromosomally unstable and exhibit a dramatic increase in whole chromosome aneuploidies. Our results thus reveal an unexpected mechanism by which tetraploidy drives CIN, in which the acquisition of chromosomally-unstable microtubule dynamics contributes to chromosome segregation errors following tetraploidisation.

## Abstract (web summary)

During cell division, tetraploidy can drive chromosomal instability (CIN) via supernumerary centrosomes, but it is unclear if this is the only route to CIN. Here the authors show that, in early mouse embryos, tetraploidy can drive chromosomal instability by altering microtubule dynamics and attachment.

## Introduction

Cell division is comprised of mitosis and cytokinesis. During mitosis, a bipolar spindle is organised by two centrosomes, each comprising a pair of centrioles surrounded by the pericentriolar material. The spindle segregates sister chromatids by the attachment of kinetochores to microtubules that generate forces to separate the chromatids^1,2^. Following chromosome alignment in metaphase, sister chromatids are segregated during anaphase. In most cells, cytokinesis occurs concomitantly causing the cytoplasm to be partitioned into two daughter cells to house the newly segregated chromosomes^3,4^. Defects in either process can affect genetic fidelity. Whereas chromosome mis-segregation in mitosis can cause gains or losses of whole chromosomes, termed aneuploidy, cytokinesis failure leads to an entirely duplicated genome, termed tetraploidy^5,6^.

Importantly, tetraploidy can trigger persistent chromosomal mis-segregation (also known as chromosomal instability; CIN), and therefore drive aneuploidy^5,6^. Indeed, tetraploidy is considered a common steppingstone in tumorigenesis and likely contributes to the high levels of CIN in cancer^5,7–11^. Landmark studies described a mechanism underpinning this phenomenon, wherein the excess of centrioles generated by failed cytokinesis causes multipolar spindles during subsequent mitoses. These multipolar spindles can cluster their extra centrosomes to form a bipolar spindle prior to anaphase, but in doing so increase the likelihood of segregation error and whole-chromosome aneuploidy^9,11–13^. Whether this is the only mechanism by which tetraploidy promotes CIN is unknown.

The early mouse embryo lacks centrioles. Whereas in most mammals the fertilising sperm provides the centrioles^14^, in mouse they are eliminated both in the oocyte and the sperm, such that the first several mitoses occur in the complete absence of centrioles, and new centrioles are eventually manufactured de novo in the ~64-cell stage embryo^15–17^. Here we take advantage of this highly unusual scenario to investigate the impact of tetraploidy upon chromosome segregation in an acentriolar setting. By extensive live time-lapse imaging we show that, in the acentriolar mouse embryo, tetraploidy rapidly leads to CIN by a mechanism independent of supernumerary centrosomes.

## Results

### Tetraploid mouse embryos are highly chromosomally unstable

**Fig. 1:**
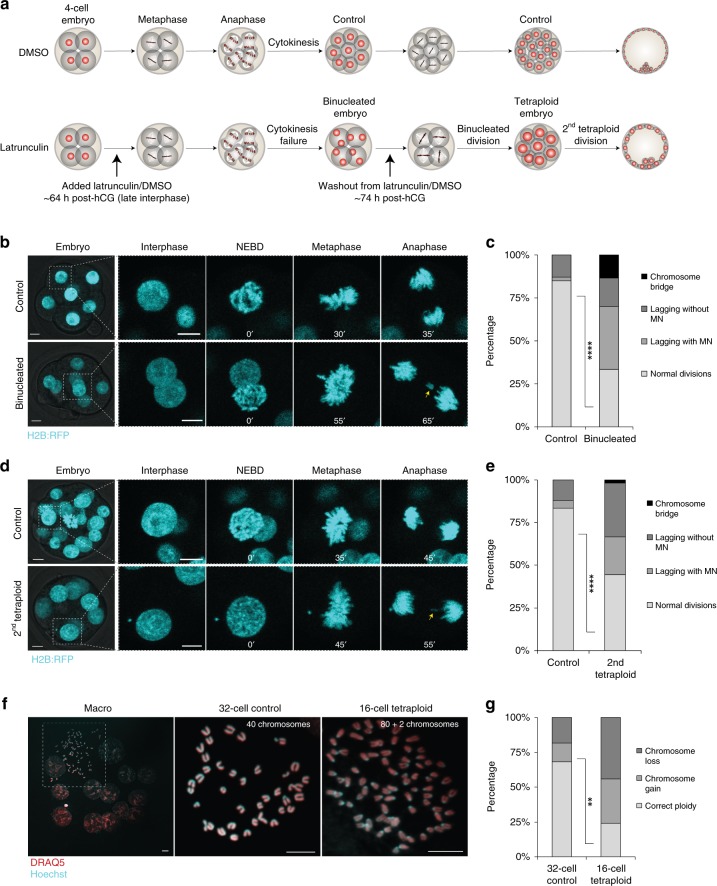
Tetraploidy leads to chromosomal instability and aneuploidy in the mouse embryo. **a** Scheme illustrating the experimental design applied for the generation of tetraploid embryos. **b** and **d** Representative time-lapse images of mitosis in live H2B:RFP-expressing 8-cell control and 4-cell binucleated **b**, 16-cell control and 8-cell tetraploid embryos **d**. A lagging chromosome (yellow arrows) can be observed both in the binucleated and second tetraploid divisions. **c** and **e** Percentage of cell divisions containing chromosome segregation errors in 8-cell control (*n* = 47 divisions from seven embryos), 4-cell binucleated embryos (*n* = 30 divisions from eight embryos) ****P < 0.0001 (two-tailed Fisher’s exact test) **c**; 16-cell control (*n* = 66 divisions from seven embryos) and 8-cell tetraploid embryos (*n* = 50 divisions from nine embryos) ****P < 0.0001 (two-tailed Fisher’s exact test) **e**. Chromosome segregation errors observed included: lagging chromosomes resulting in micronuclei formation (lagging with MN); lagging chromosomes that did not result in micronuclei formation (lagging without MN); and chromosome bridges. **f** Representative images of chromosome spreads obtained from 32-cell control and 16-cell tetraploid embryos. **g** Percentage of blastomeres containing whole chromosome gains and losses in 32-cell control (*n* = 22 spreads) and 16-cell tetraploid embryos (*n* = 25 spreads) **P = 0.0034 (two-tailed Fisher’s exact test). Scale bars = 10 µm. NEBD nuclear envelope breakdown

To explore the impact of tetraploidy, we transiently prevented cytokinesis using the actin depolymerising agent Latrunculin B at the 4–8-cell transition, thereby obtaining embryos with four binucleated blastomeres (Fig. 1a, see also the section “Methods”). Herein we refer to the next cell division, in which the four binucleated blastomeres divide to become eight mono-nucleated blastomeres as the ‘binucleated division’, and the subsequent division as the ‘second tetraploid division’ (Fig. 1a). In some somatic cells, tetraploidy results in a p53-dependent cell cycle checkpoint that prevents further cell division^18,19^. To assess the impact of tetraploidy in embryos, we allowed binucleated embryos to develop in vitro and counted cell numbers 12 and 24 h after binucleation using fixed-cell analysis. Embryos developed from the binucleated four-cell stage to become morulae possessing 17.9 ± 1.15 (mean ± SEM, *n* = 23 embryos) cells 24 h after binucleation, confirming that cell divisions were not critically impeded (Supplementary Fig. 1a, b). Next, we used PCNA:EGFP to visualise cell cycle progression^20^. Nuclear PCNA:EGFP foci were transiently evident in mid-interphase both in the binucleated division and the second tetraploid division, similar to control embryos, indicative of successful progression through S phase (Supplementary Fig. 1c, d). Accordingly, the number of kinetochores, as observed by kinetochore immunostaining in metaphase-arrested embryos, was doubled in Latrunculin-treated embryos as compared to controls (Supplementary Fig. 1e, f). Thus, as suggested previously^21–23^, preimplantation mouse embryos fail to mount a tetraploidy-induced cell cycle checkpoint and continue to develop with a doubled genome.

We wondered about the impact of tetraploidy upon chromosome segregation in embryos. Analysis of chromosome segregation dynamics in live H2B:RFP-expressing embryos at the binucleated division revealed that whereas 82% of divisions in control embryos were normal and without obvious defects, segregation defects were common in tetraploid embryos, with only 33% of divisions occurring with no observable defect (Fig. 1b, c). Embryos also displayed segregation defects when cytokinesis failure had been induced with either Cytochalasin B or Blebbistatin (Supplementary Fig. 2a, b). Moreover, embryos undergoing mitosis in the continued presence of the actin inhibitor Latrunculin had few errors (Supplementary Fig. 2c–e), confirming that actin depolymerisation does not negatively affect chromosome segregation fidelity in the mouse embryo, contrary to the case of mouse oocyte meiosis I^24^. Together, these experiments confirm that the errors observed are attributable to the tetraploid state of the embryos, not the method of inducing tetraploidy. Strikingly, analysis of the second tetraploid division revealed a phenotype very similar to that of the binucleated division, whereas stage-matched controls showed few discernible defects (Fig. 1d, e). Increased abundance of micronuclei, a marker of accumulated chromosome segregation errors^25^, was also observed in fixed cell experiments, excluding the possibility that the increased number of errors was somehow related to live imaging (Supplementary Fig. 3). Importantly, ploidy analysis by chromosome spreads at early blastocyst stage revealed that 68.2% of 32-cell stage control embryos contained 40 chromosomes, whereas 31.8% had either chromosome gains or losses (Fig. 1f, g), with chromosome numbers ranging from 38 to 42 (Supplementary Fig. 4a). In contrast, we found that only 24% of 16-cell stage tetraploid embryos maintained a perfect tetraploid genome (80 chromosomes) (Fig. 1f, g), with chromosome numbers ranging from 77 to 83 (Supplementary Fig. 4b). Taken together these experiments reveal that tetraploid mouse embryos continue to divide but become chromosomally unstable during the next few cell divisions.

### CIN is not attributable to supernumerary centrosomes

**Fig. 2:**
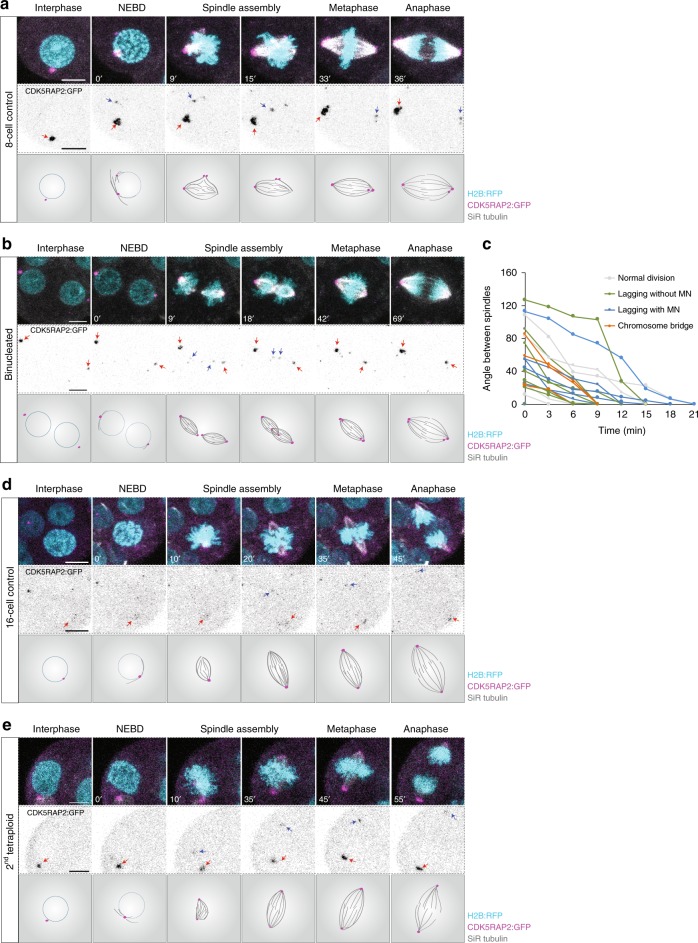
Chromosomal instability in tetraploid embryos is not attributable to supernumerary centrosomes. **a**, **b**, **d** and **e** Representative time-lapse images and illustrations of live 8-cell control (**a**), 4-cell binucleated (**b**), 16-cell control (**d**) and 8-cell tetraploid embryos (**e**) labelled with SiR Tubulin (grey) and co-expressing H2B:RFP (cyan) and CDK5RAP2:GFP (magenta and inverted grey). A major microtubule organising centre (MTOC) can be observed in the 8-cell control, 16-cell control and 8-cell tetraploid embryo during interphase (red arrows) and a newly assembled MTOC can be observed during mitosis (blue arrows). In binucleated embryos, two major MTOCs (red arrows) can be observed during interphase and two newly assembled MTOCs (blue arrows) can be observed during mitosis. **c** Measurements of the angle between the two individualised spindles during the binucleated division. Line colours represent the different types of chromosome segregation events associated with a specific cell division (*n* = 23 divisions from 12 embryos). Chromosome segregation errors observed included: lagging chromosomes resulting in micronuclei formation (lagging with MN); lagging chromosomes that did not result in micronuclei formation (lagging without MN); and chromosome bridges. Scale bars = 10 µm. NEBD nuclear envelope breakdown

In tetraploid somatic cells caused by cytokinesis failure, supernumerary centrosomes lead to the formation of transiently multipolar spindles that promote segregation errors^9,11^. Preimplantation mouse embryos lack centrioles until ~64-cell stage but achieve spindle assembly between the 4-cell and 32-cell stage by acentriolar microtubule-organising centres (MTOCs)^26^. We therefore set out to simultaneously observe MTOCs, spindles, and chromosome dynamics in tetraploid embryos, using CDK5RAP2:GFP^27,28^, SiR Tubulin^29^ and H2B:RFP, respectively. During interphase, the majority of normal diploid 8-cell embryos displayed a single clear MTOC close to the nucleus (Fig. 2a; Supplementary Fig. 5c and Supplementary Movie 1). At the onset of nuclear envelope breakdown (NEBD) a new MTOC was assembled such that most diploid embryos displayed a clear CDK5RAP2:GFP-labelled MTOC at each spindle pole at metaphase (Fig. 2a; Supplementary Fig. 5d and Supplementary Movie 1)^16,17^. Analogously, during the binucleated division, 4-cell binucleated embryos typically displayed a single clear MTOC on each nucleus during interphase (Fig. 2b; Supplementary Fig. 5a and c; Supplementary Movie 2). Shortly after NEBD, two new MTOCs were usually assembled such that most binucleated embryos displayed four MTOCs during mitosis (Fig. 2b and Supplementary Fig. 5a, d; Supplementary Movie 2). Interestingly, these four MTOCs usually formed the poles of two completely separate bipolar spindles (Fig. 2b and Supplementary Fig. 5a, b; Supplementary Movies 2, 3). These two spindles rapidly moved towards each other and fused to form a single bipolar spindle prior to anaphase (Supplementary Movies 2, 3). We measured and tracked the angles between the two spindles from the moment they first established contact until they completely fused (Fig. 2c). Upon contact, the spindles fused by either sliding together or rotating towards each other depending on the initial angle of contact until they eventually became a single bipolar spindle (Fig. 2b, c; Supplementary Fig. 5a, b; Supplementary Movies 2, 3), as observed in Fmn2^−/−^ mouse oocytes^30^, Xenopus extract spindles in close apposition^31^, and in mouse zygotes^32^. Notably however, there was no relationship between the initial angle at which the spindles made contact with each other and the likelihood of developing chromosome segregation errors (Fig. 2c). Moreover, multipolar spindles such as are characteristic in somatic cells with supernumerary centrioles, and associated with segregation error, were rare both in 4-cell binucleated (18.4% of divisions) and 8-cell control embryos (11.4% of divisions). Importantly, we used 3-min acquisitions intervals, which allowed us to confidently distinguish between spindle fusion events and multipolarity. Notably, even in more extreme examples of perpendicular spindle fusion, the two spindles remained distinguishable throughout fusion without neighbouring poles connecting via microtubule bundles (Supplementary Fig. 5a, b; Supplementary Movie 3).

Next, we analysed spindle dynamics in the second tetraploid division which, importantly, begins with a single morphologically normal nucleus containing a tetraploid genome. Similar to 8-cell and 16-cell controls, these cells usually possessed only one major MTOC adjacent to the nucleus and a second major MTOC was assembled at NEBD to enable the generation of a bipolar spindle (Fig. 2d, e; and Supplementary Fig. 5e, f). Though general spindle morphology was not obviously altered as compared to diploid controls, tetraploid blastomeres possessed a wider metaphase plate, presumably as a result of having twice as many chromosomes (Supplementary Fig. 5g, h). Spindle assembly was otherwise morphologically indistinguishable from diploid 8-cell or 16-cell embryos, despite the dramatic increase in segregation errors (Fig. 1d, e), with multipolar spindles again very rare (16-cell control: 9.6% of divisions; 8-cell tetraploid: 16.6% of divisions). Taken together, these observations reveal that CIN observed in the tetraploid mouse embryo cannot be attributed to supernumerary centrioles or multipolar spindles.

### Spindle assembly checkpoint (SAC) is not abrogated by tetraploidy

**Fig. 3:**
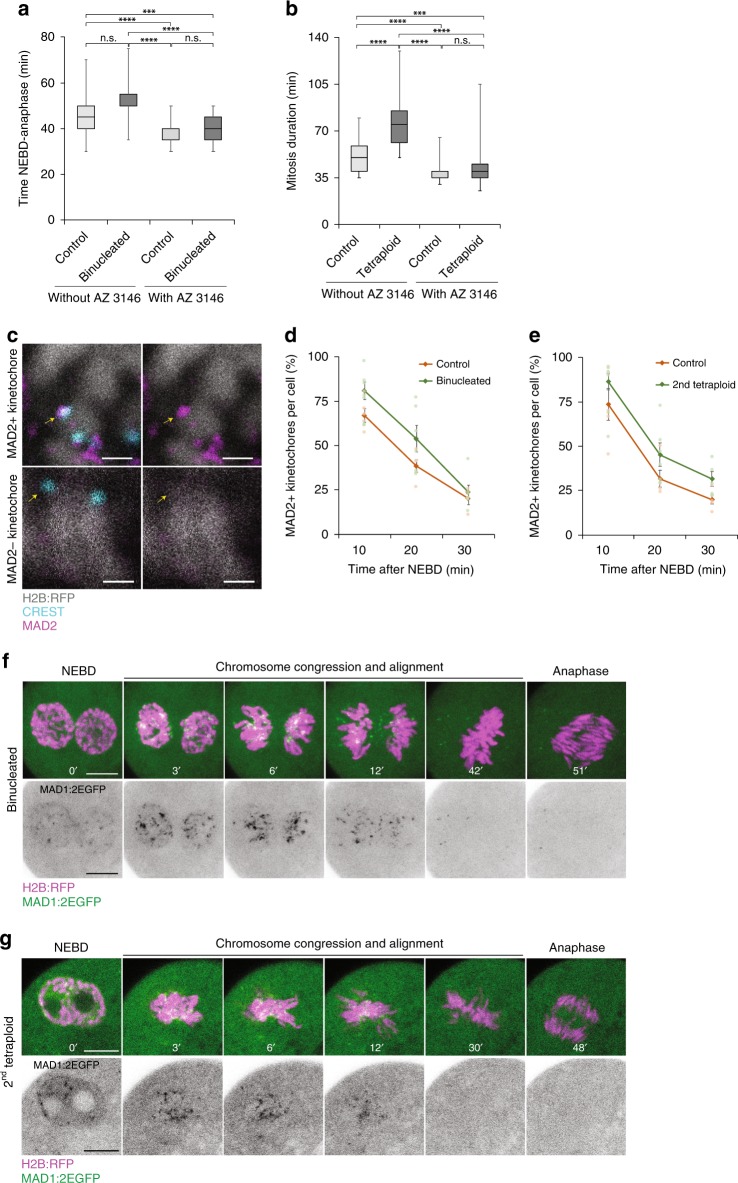
Spindle assembly checkpoint activity is not abrogated in tetraploid embryos. **a** and **b** Quantification of mitosis duration in 8-cell control (without AZ 3146 *n* = 47 divisions from seven embryos; with AZ 3146 *n* = 63 divisions from eight embryos), 4-cell binucleated embryos (without AZ 3146 *n* = 30 divisions from eight embryos; with AZ 3146 *n* = 54 divisions from 15 embryos) ***P = 0.0009; ****P < 0.0001 (unpaired Kruskal–Wallis test with Dunn’s test for multiple comparisons) **a**; 16-cell control (without AZ 3146 *n* = 61 divisions from seven embryos; with AZ 3146 *n* = 59 divisions from seven embryos) and 8-cell tetraploid embryos (without AZ 3146 *n* = 50 divisions from nine embryos; with AZ 3146 *n* = 63 divisions from 12 embryos) ***P = 0.0005; ****P < 0.0001 (unpaired Kruskal–Wallis test with Dunn’s test for multiple comparisons) **b**. **c** Representative z projections of MAD2-positive and MAD2-negative kinetochores demonstrating co-localisation of MAD2 (magenta) and CREST (cyan) in positively stained kinetochores and no co-localisation in negatively stained kinetochores. **d** and **e** Proportion of MAD2-positive kinetochores in 8-cell control, 4-cell binucleated **d**, 16-cell control and 8-cell tetraploid embryos **e** at 10 mins (8-cell control *n* = 6 blastomeres; 4-cell binucleated *n* = 6 blastomeres; 16-cell control *n* = 5 blastomeres; 8-cell tetraploid *n* = 5 blastomeres), 20 mins (8-cell control *n* = 5 blastomeres; 4-cell binucleated *n* = 6 blastomeres; 16-cell control *n* = 5 blastomeres; 8-cell tetraploid *n* = 6 blastomeres) and 30 mins (8-cell control *n* = 4 blastomeres; 4-cell binucleated *n* = 6 blastomeres; 16-cell control *n* = 5 blastomeres; 8-cell tetraploid *n* = 5 blastomeres) after nuclear envelope breakdown (NEBD). Error bars represent SEM. **f** and **g** Representative time-lapse images of live 4-cell binucleated **f** and 8-cell tetraploid embryos **g** co-expressing H2B:RFP and MAD1:2EGFP. Note that MAD1:2EGFP signal is clearly observed at the kinetochores shortly after nuclear envelope breakdown (NEBD) and is gradually lost following chromosome alignment at the metaphase plate. Scale bars = 10 µm except for figure **c**, where scale bars = 1 µm. Where box plots are shown, the centre line represents the median, the bounds of box represent the upper and lower quartiles and the whiskers represent minimum and maximum values

Most cells possess a signalling pathway termed the SAC that serves to prevent chromosome segregation errors by delaying anaphase until all chromosomes are aligned at the metaphase plate and the kinetochores attached to microtubules^33,34^. Notably, recent work has shown that, in early embryos, although misaligned chromosomes/kinetochores also recruit SAC proteins, such as Mad1 and Mad2, SAC signalling is not sufficiently robust to enforce a metaphase arrest^35–37^. We wondered whether the higher rates of error in tetraploid embryos might be attributable to further weakening of the SAC, and thus set out to probe SAC activity in tetraploid embryos. Notably, tetraploidy prolonged mitosis, as observed by increased NEBD-anaphase duration (Fig. 3a, b), suggesting SAC activation. SAC inhibition using the Mps1 inhibitor AZ 3146 reduced the duration of mitosis causing controls and tetraploids to have a similar M-phase duration, indicating that the prolonged mitosis in tetraploids was attributable to the SAC (Fig. 3a, b). Immunofluorescence analysis of the SAC protein Mad2 revealed that the majority of kinetochores exhibited pronounced Mad2 staining shortly after NEBD which was lost as chromosomes aligned, similar to controls (Fig. 3c–e). Similarly, live imaging of tetraploid embryos co-expressing H2B:RFP and MAD1:2EGFP clearly shows MAD1:2EGFP recruitment to kinetochores shortly after NEBD and gradual loss of signal within 30–40 min as chromosomes align at the metaphase plate (Fig. 3f, g). As is the case in normal diploid mouse embryos^36^, tetraploid blastomeres frequently failed to wait for full chromosome alignment prior to anaphase onset, underscoring the previously reported inefficiency of SAC in the mouse embryo (Supplementary Fig. 6). Nonetheless, tetraploidy caused a substantial SAC-dependent lengthening of mitosis. Therefore, though we cannot exclude the possibility of minor impacts of tetraploidy upon the SAC, our experiments fail to uncover a clear weakening of the SAC in tetraploids that might explain the high rate of segregation error.

### Tetraploidy perturbs metaphase chromosome alignment

**Fig. 4:**
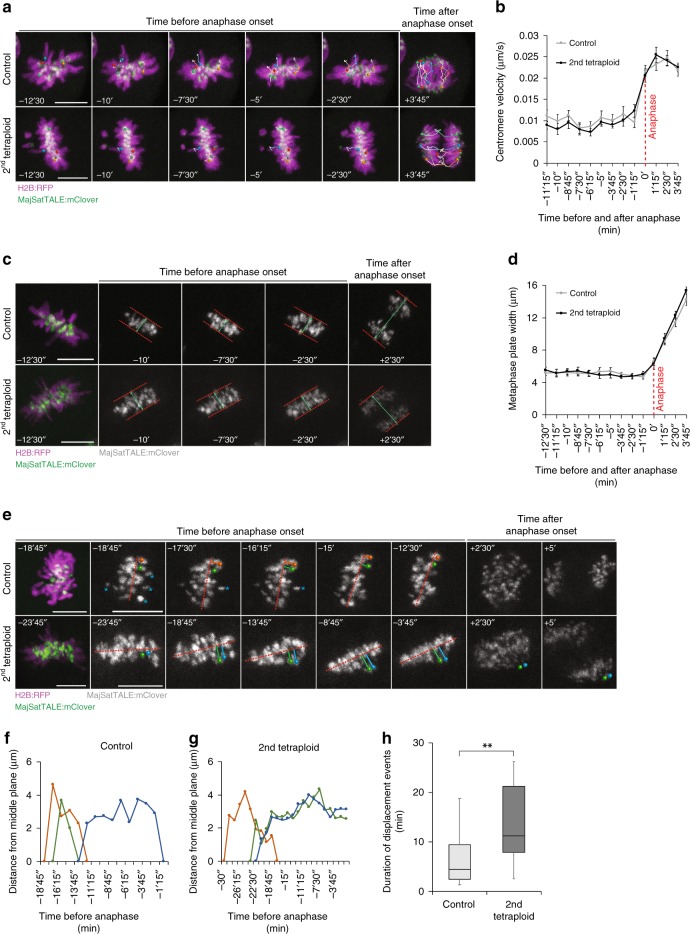
Tetraploid embryos exhibit chromosome alignment defects. **a** Representative time-lapse images of centromere tracking in 16-cell controls and 8-cell tetraploid embryos co-expressing H2B:RFP and MajSatTALE:mClover. Coloured circles indicate centromere pairs that were tracked throughout mitosis and individualised centromeres at anaphase. Coloured lines represent the path covered by the centromere pairs during time. **b** Quantification of average centromere velocity in 16-cell control (*n* = 24 centromere pairs from five blastomeres) and 8-cell tetraploid embryos (*n* = 21 centromere pairs from five blastomeres). **c** Representative time-lapse images of 16-cell control and 8-cell tetraploid embryos co-expressing H2B:RFP and MajSatTALE:mClover, demonstrating the measurements of metaphase plate width throughout mitosis. Red dashed lines indicate the borders of the metaphase plate based on the MajSatTALE:mClover signal. Green lines indicate width measurements. **d** Quantification of metaphase plate width in 16-cell control (*n* = 5 blastomeres) and 8-cell tetraploid embryos (*n* = 5 blastomeres). **e** Representative time-lapse images of 16-cell control and 8-cell tetraploid embryos co-expressing H2B:RFP and MajSatTALE:mClover, demonstrating chromosome displacement events. In the control embryo, two chromosomes (orange and green circles—corresponding to the orange and green lines in figure **f**) that were previously aligned become displaced from the middle plane of the metaphase plate (red dashed lines) at mid-mitosis, returning to their original position before anaphase onset. In the tetraploid embryo, the two chromosomes that become displaced (green and blue circles—corresponding to the green and blue lines in figure **g**) fail to return to the metaphase plate, resulting in misaligned chromosomes at anaphase onset. Blue asterisks indicate chromosomes that were not yet aligned for the first time during the time sequence shown. **f** and **g** Quantification of the distance between centromere pairs and metaphase middle plane in a 16-cell control **f** and 8-cell tetraploid embryo **g**. **h** Quantification of average duration of displacement events in 16-cell controls (*n* = 16 displacement events from five blastomeres) and 8-cell tetraploid embryos (*n* = 14 displacement events from five blastomeres; **P = 0.0061, unpaired two-tailed Mann–Whitney test). In the box plot, the centre line represents median, the bounds of box represent upper and lower quartiles and the whiskers represent minimum and maximum values. Scale bars = 10 µm. Error bars represent SEM

To further investigate how tetraploidy leads to lagging chromosomes and chromosome segregation error, we performed a comprehensive analysis of centromere spatiotemporal behaviour in mitosis. Embryos co-expressing H2B:RFP and the centromere label MajSatTALE:mClover^38^ were imaged in three dimensions at 75 s intervals, and individual centromere pairs were tracked during the second tetraploid division. Our analysis showed no difference in centromere velocity either prior to anaphase onset or during anaphase poleward chromosome movement (Fig. 4a, b), or in the tightness of the metaphase plate (Fig. 4c, d), suggesting that overall behaviour of most chromosomes is not adversely affected in tetraploids. Interestingly, however, we observed that both controls and tetraploid blastomeres displayed chromosomes that, having previously been aligned, displaced from the metaphase plate to become unaligned (Fig. 4e). In controls, these displacement events lasted on average 6.32 ± 1.43 min (*n* = 16 displacement events; Fig. 4h) during which time the centromeres typically moved between 1.7 and 10.74 µm from the metaphase plate, before returning to full alignment. Importantly, in control blastomeres, chromosomes that became displaced from the metaphase plate returned to full alignment prior to anaphase onset in almost all cases (Fig. 4e, f and h). On the other hand, in tetraploid embryos, chromosome displacement events lasted substantially longer (13.57 ± 2.11 min, *n* = 14 displacement events), and in many cases chromosomes failed to completely re-align prior to anaphase onset (Fig. 4e, g, h). Of the misaligned chromosomes at anaphase onset observed in tetraploid embryos, almost all resulted from a chromosome that was previously aligned and became displaced during metaphase. Consistent with our previous results, the majority of tetraploid embryos displayed lagging chromosomes. Importantly, all anaphase laggards arose from previously metaphase-aligned chromosomes, revealing that the elevated frequency of lagging chromosomes in tetraploid embryos is not attributable to failed alignment (Supplementary Fig. 7).

Taken together, these results show that, though overall centromere behaviour and chromosome congression are largely unaltered in tetraploid embryos, a deficiency in maintaining chromosome alignment leads to an increased likelihood of chromosome misalignment at anaphase. However, these misaligned chromosomes do not seem to be the major cause of lagging chromosomes in tetraploid embryos. Rather, similar to somatic^39^ and cancer cells^40^ lagging chromosomes arise from chromosomes that were correctly aligned at the metaphase plate prior to anaphase onset.

### Tetraploidy affects kinetochore microtubule establishment

**Fig. 5:**
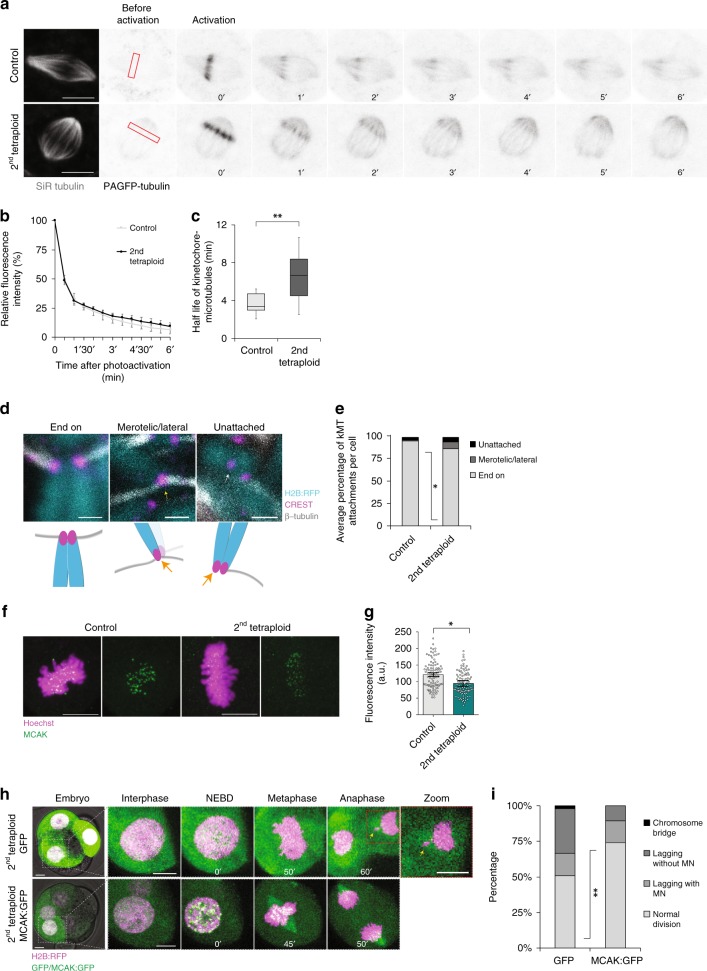
Error correction mechanisms are defective in tetraploid embryos. **a** PAGFP-tubulin (inverted grey) was photoactivated at a rectangular region across one side of the metaphase spindle (red rectangles) and the time-course for fluorescence decay was monitored. Embryos were labelled with SiR Tubulin (grey) to identify the metaphase spindle. **b** and **c** Quantification of fluorescence decay values **b** and kinetochore–microtubule half-life (**c**; **P = 0.008, unpaired, two-tailed *t* test) in 16-cell control (*n* = 11 blastomeres) and 8-cell tetraploid embryos (*n* = 9 blastomeres). In the box plot, the centre line represents median, the bounds of box represent upper and lower quartiles and the whiskers represent minimum and maximum values. **d** Representative z-projections of end-on, merotelic/lateral, and unattached kinetochores at 35 min after nuclear envelope breakdown (NEBD); and scheme illustrating the types of kinetochore–microtubule attachments analysed. Representative images were chosen to demonstrate the different types of attachments. For the merotelic/lateral attachment image, only the merotelically attached kinetochore is in focus (as represented in the illustration by a fainter colour of the sister chromatid). **e** Average percentage of end-on, merotelic/lateral and unattached kinetochores per cell in 16-cell (*n* = 189 kinetochores from five blastomeres) control and 8-cell tetraploid embryos (*n* = 223 kinetochores from five blastomeres; *P = 0.0148, unpaired, two-tailed *t* test). **f** Representative z-projections of MCAK immunofluorescence in 16-cell control and 8-cell tetraploid embryos. **g** Quantification of fluorescence intensity in 16-cell control and 8-cell tetraploid embryos (*n* = 100 kinetochores from 10 blastomeres per group). Statistical analysis was performed as an inter-embryo comparison using the average from 10 individual kinetochores analysed per blastomere, *P = 0.0223 (unpaired, two-tailed *t* test). **h** Representative time-lapse images of 8-cell tetraploid embryos co-expressing H2B:RFP and either GFP or MCAK:GFP. The yellow arrow indicates a lagging chromosome. **i** Percentage of cell divisions displaying chromosome segregation errors in 8-cell tetraploid embryos co-expressing H2B:RFP and either GFP (*n* = 51 divisions from 16 embryos) or MCAK:GFP (*n* = 66 divisions from 23 embryos) **P = 0.0093 (two-tailed chi-square). Chromosome segregation errors observed included: lagging chromosomes resulting in micronuclei formation (lagging with MN); lagging chromosomes that did not result in micronuclei formation (lagging without MN); and chromosome bridges. Scale bars = 10 µm, except for **d**, where scale bars = 1 µm. Error bars represent SEM

During spindle assembly some kinetochore pairs form merotelic attachments in which a single kinetochore simultaneously contacts microtubules (MTs) from both spindle poles. Establishment of correct (amphitelic) chromosome attachment prior to anaphase occurs by correction of previously mis-attached kinetochores, a process that depends upon MT turnover at the kinetochore^41,42^. In cancer cells, CIN is associated with reduced kinetochore–microtubule (kMT) turnover that promotes errors including merotelic attachments that lead to lagging chromosomes and aneuploidy^43^. We directly analysed kMT turnover during the second tetraploid division using fluorescence dissipation after photoactivation of photoactivatable-GFP-tubulin (PAGFP-tubulin) within the metaphase spindle as previously described^43–45^, using SiR Tubulin to select cells with spindles oriented in the plane of imaging (Fig. 5a–c). Strikingly, kMT half-life was substantially increased in tetraploid embryos compared to controls (Fig. 5a–c), indicating reduced kMT turnover. Notably, this increase is similar to that observed when comparing chromosomally unstable to chromosomally stable somatic cells^43^. Non-kinetochore MT half-life and the rate of poleward microtubule flux measured with PAGFP-tubulin, as well as the velocity of microtubule growth events as determined by EB1:EGFP imaging, were all unchanged between tetraploids and controls (Supplementary Fig. 8a–e), revealing that the change in MT dynamics was highly specific to kMT turnover. Importantly, there was no difference in MT turnover when comparing SiR Tubulin-stained blastomeres with SiR Tubulin-free blastomeres, ruling out the possibility that the increase in kMT half-life observed in tetraploid embryos could have been induced by potential MT-stabilising effects of SiR Tubulin (Supplementary Fig. 8f–h). Highly consistent with a kMT turnover defect, direct comparison of stable kMT attachments in embryos with a classic cold shock approach revealed a far greater proportion of mis-attachments in tetraploid embryos as compared to controls in mid-late prometaphase/metaphase (Fig. 5d, e). Whereas lateral/merotelic attachments were very rare in diploid blastomeres (0.9%), 7% of all attachments observed in tetraploid blastomeres were misattached, at least one kinetochore being misattached in every tetraploid blastomere examined. This is highly consistent with the notion that a single merotelically attached kinetochore is sufficient to cause missegregation^39^ and correlates with the elevated rates of lagging chromosomes observed in tetraploids (Fig. 1b–e).

A major determinant of the capacity to maintain kinetochore MT turnover to correct misattachments is the recruitment of the microtubule depolymerising protein mitotic centromere-associated kinesin (MCAK)^46,47^. We wondered whether MCAK was sufficiently recruited to the centromere/kinetochore in tetraploid embryos. Using immunofluorescence we found that MCAK was significantly underrepresented at the kinetochore in tetraploid embryos as compared to controls (Fig. 5f, g). This suggests that reduced MT turnover may be downstream of an inability to recruit sufficient MT-turnover-sustaining factors. Consistent with this notion, overexpression of MCAK:GFP substantially and significantly decreased the rates of chromosome segregation errors in tetraploid embryos, when compared to tetraploid embryos expressing GFP alone (Fig. 5h, i), as has also been seen in cancer cells ectopically expressing MCAK^48^. Taken together these data show that, in tetraploid embryos, kMT turnover is reduced and error correction mechanisms are suppressed, allowing for the accumulation of merotelic attachments that in turn lead to lagging chromosomes, which can be rescued by introducing ectopic MCAK:GFP.

## Discussion

Our data show that mouse embryos do not possess a tetraploidy checkpoint but continue to divide and become chromosomally unstable immediately after tetraploidisation (Fig. 1). Why the mouse embryo fails to mount a tetraploidy checkpoint is unclear but may relate to the key role of centrioles in mediating the checkpoint, shown in RPE1 cells^19^. The described mechanism by which tetraploidy leads to CIN in mammalian cells is that the acquisition of supernumerary centrioles/centrosomes leads to the formation of hazardous multipolar spindles that induce segregation errors^5,7,9,11^. That there might be other means by which tetraploidy could cause CIN was alluded to in studies of tetraploid yeast with normal centrosome numbers, where CIN was likely due to the impact of cell size upon spindle geometry^49^. Here we show that mouse blastomeres, which are acentriolar, become highly chromosomally unstable upon tetraploidisation as a result of altered kMT dynamics. Notably, this mechanism is distinct to yeast, where tetraploid spindles have unchanged microtubule dynamics^49^. Our data indicate that reduced kinetochore recruitment of MCAK, a well-characterised MT depolymerising kinesin, provides at least part of the explanation for the reduced kinetochore MT turnover that underpins misattachments and segregation errors in tetraploid blastomeres. Why MCAK is underrepresented at the kinetochores remains to be determined. Gene expression changes associated with having extra copies of chromosomes^50^, and the doubling of kinetochores, could present an overburden on the ability to recruit MCAK. Alternatively, tetraploidy could impact upstream signalling necessary for MCAK recruitment to the kinetochore^51,52^, or affect the structure of the kinetochore itself.

Our centromere and chromosome tracking data show that tetraploidy introduces two types of anaphase defects. First, we observed lagging anaphase chromosomes, that arise from fully aligned metaphase chromosomes, and frequently result in a micronucleus. Whereas in somatic cells micronuclei derived from lagging chromosomes are frequently reabsorbed in the next cell cycle, which likely forms the mechanistic basis for chromothripsis^53^, we have previously shown that in mouse embryos micronuclei are very rarely reincorporated into the principal nucleus, and repeated micronucleus inheritance necessarily drives aneuploidy^25^. Notably, micronuclei are also unilaterally inherited in tetraploid embryos (Supplementary Fig. 9), thus lagging chromosomes seem certain to contribute to mosaic aneuploidy in the mouse tetraploid embryo. Secondly, we observe chromosomes that fail to align in time for anaphase onset. In some cases, the proximity of the misaligned chromosome to the metaphase plate, paired with imaging resolution limits prevented us from determining whether the two sister chromatids were ultimately correctly segregated. However, in at least one example where we labelled both the chromosome and the centromere in live imaging, both misaligned sister chromatids were seen to migrate to the same spindle pole (Supplementary Fig. 7). Thus, although formally distinguishing the proportional contribution of these two distinct defects to embryo aneuploidy is experimentally challenging, both likely contribute to the elevated rate of aneuploidy in the tetraploid embryo.

Tetraploidy is common in the early stages of tumorigenesis, and likely contributes to the CIN and aneuploidy associated with some cancers^5–9,54^. Genome doubling is thought to provide a permissive environment for the acquisition of CIN, as chromosome losses are more likely to be tolerated with multiple chromosome copies present^54^. Importantly, our data do not oppose the notion that centriole doubling can be a major driver of CIN, but rather add altered microtubule dynamics as a distinct mechanism that can also confer CIN immediately after tetraploidisation. In tetraploid somatic cells, the extra centrioles are lost after repeated passages^9,55^, and it remains to be seen whether an analogous adaptation might occur with microtubule dynamics. Nonetheless our data adds altered MT dynamics to supernumerary centrosomes as two separate defects that emerge rapidly to drive CIN in the first cell cycles following tetraploidisation.

## Methods

### Embryo culture and microinjection

All experiments were performed in accordance with the guidelines for animal experimentation of the Comité Institutionnel de Protections des Animaux (CIPA). All experiments were approved by the Centre de Recherche du Centre Hospitaliaire de l’Université de Montréal (CRCHUM) Comité Institutionnel de Protections des Animaux (CIPA). Protocol number: IP18034GFs. Embryos were harvested from superovulated CD1 female mice (Crl:CD1(ICR) Charles River Laboratories) mated with CD1 male mice, and cultured in KSOM medium (EmbryoMax^®^ KSOM; Millipore, MR-020P-5F) in 5% CO_2_ at 37 °C. mRNA was manufactured using Ambion mMessage Machine T3 (AM1348) or T7 (AM1344) according to manufacturer’s instructions. Plasmids used were H2B:RFP in pRN4 (gift from Alex McDougall), PCNA:EGFP in pcDNA3.1 + poly(A) (gift from Kazuo Yamagata), CDK5RAP2:GFP in pGEMHE (gift from Tomoya Kitajima), α-tubulin-human:PAGFP in pIRESHyg2 (Addgene, plasmid #12296), EB1:EGFP in pcDNA3.1 + poly(A) (gift from Lynne Cassimeris), MAD1:2EGFP in pIVT (gift from Michael Lampson), MajSatTALE:mClover in pTALYM3 (Addgene, plasmid #47878) and MCAK:GFP (purchased in the pEGFP-C1 vector from Addgene, #pYOY152 and subcloned into pcDNA.3.1/myc-His(–)A). Two-cell embryos were microinjected using a picopump (World Precision Instruments) and micromanipulators (Narishige) mounted on a Leica DMI4000 inverted microscope^45^. For the experiments in Fig. 3f, the embryos were microinjected at the 4-cell stage. For the experiments in Figs. 3g and 5h, the embryos were microinjected at late 4-cell binucleated stage (equivalent to 8-cell stage).

### Drug treatments

To induce binucleation, late interphase 4-cell stage embryos (~64 h post-hCG) were treated for ~10 h with Latrunculin B (5 µM; EMD Millipore,428020). Embryos that did not display the four blastomeres with two visible nuclei by the end of the incubation period were excluded. Simultaneously, control embryos were treated with 1:1000 DMSO (Sigma Aldrich, D2650). After the incubation period, the embryos were thoroughly washed and cultured in KSOM media. For experiments in Supplementary Fig. 2a, b, either Cytochalasin B (5 µg/mL; Sigma-Aldrich C6762) or Blebbistatin (100 µM; Calbiochem/Millipore, 203391) were used to induce binucleation. For experiments in Supplementary Figs. 1e, f,  5g, h; Figs. 1f, g; 5f, g, the embryos were treated with 25 µM of MG 132 (Calbiochem 474790) to induce a metaphase arrest. For kinetochore counts in Supplementary Fig. 1e, f, after metaphase arrest the embryos were treated with 200 µM of Monastrol (Calbiochem, 475879) to induce monopolar spindle formation and allow better visualisation of individual kinetochores. For the SAC inhibition treatment in Fig. 3a, b, the embryos were live-imaged in the presence of AZ 3146 (Calbiochem, catalogue #531976). For experiments involving PAGFP:tubulin live imaging, embryos were arrested in metaphase using 100 µM of APCin (Tocris, 5747). For the photobleaching control experiments, MII eggs were live-imaged in the presence of 10 µM of Taxol (Paclitaxel; Sigma Aldrich, T402). Spindle labelling in live embryos was performed with a 2 h incubation in 300 nM of SiR Tubulin (Cytoskeleton Inc., CY-SC002). For the experiment in Supplementary Fig. 8f–h, the embryos were either exposed to 1 µM of SiR DNA (Cytoskeleton Inc., CY-SC007) alone for 3 h or to 300 nM of SiR Tubulin for 2 h followed by a 3 h exposure to 1 µM of SiR DNA.

### Chromosome spreads

Chromosome spreads were performed using an air-drying method^56^. Metaphase arrested 32-cell diploid and 16-cell tetraploid embryos were exposed to 1% sodium citrate for 15 min and subsequently transferred to a grease-free slide. Three drops of methanol:acetic acid (3:1) were applied directly on top of the embryos and the slides were air-dried. For staining, air-dried slides were co-labelled with Hoechst 33342 (Sigma Aldrich, H6024, 1:500) and DRAQ5 (Thermo Fisher Scientific, 62254, 1:250) loaded in FluorSave™ Reagent (Calbiochem, 345789) in order to differentially label the chromosomes and centromeric regions.

### Cold shock treatment

For assessment of kMT attachments in Fig. 5d, e, H2B:RFP-expressing embryos were exposed to a 10 min cold shock treatment in ice cold M2 media (Sigma Aldrich M7167) 35 min after NEBD was observed by live imaging and then immediately fixed.

### Immunofluorescence and live Imaging

Embryos were fixed with 4% paraformaldehyde (PFA) in PBS for 40 min followed by 10 min permeabilization using 0.25% Triton X in PBS, and blocked with 3% bovine serum albumin (BSA) in PBS^57^. Where CREST antibodies were used, embryos were fixed with 2% PFA in PBS for 30 min. Where MAD2 antibodies were used, embryos were fixed with 2% PFA in PBS for 15 min. Primary antibodies used were: CREST anti-human (gift from Marvin J. Fritzler 1:100), β-tubulin anti-mouse (Sigma Aldrich catalogue #T4026 1:1000), MCAK anti-rabbit (gift from Duane Compton; 1:1000), pericentrin anti-mouse (BD Biosciences catalogue #611814 1:500), α-tubulin anti-rabbit (Abcam catalogue #AB18251 1:1000) and MAD2 anti-rabbit (Biolegend catalogue #924601; 1:300). Hoechst 33342 (1:1000) was used for DNA labelling. Alexa-labelled secondary antibodies (1:1000) were purchased from ThermoFisher.

Alexa Fluor^®^ 555 Phalloidin-conjugated antibody (1:200) was purchased from Invitrogen (A34055). Immunofluorescence imaging was performed on either a Leica SP8 confocal microscope fitted with a 63 × 1.4 numerical aperture oil objective or a Leica SP5 confocal microscope fitted with a 100 × 1.4 numerical aperture oil objective and a HyD detector. Live cell imaging and FDAP was performed on a Leica SP8 confocal microscope fitted with a 20 × 0.75 numerical aperture air objective and a HyD detector and embryos were imaged on KSOM media, placed on a heated stage top incubator with 5% CO_2_ supply at 37 °C. For the live imaging experiment performed in Supplementary Fig. 1a, the embryos were imaged in a Zeiss Axio observer, equipped with an Axiocam and Apotome and 20 × 0.8 numerical aperture air objective and LED light. For EB1:EGFP live imaging, embryos were imaged every 2.578 s for 2 min with a 63 × 1.4 numerical aperture oil objective. For live imaging in the presence of either Taxol (photobleaching control in Fig. 5a–c) or Latrunculin (Supplementary Fig. 2c, d), the embryos were live imaged in Ibidi micro-insert wells mounted in a glass-bottom dish with distilled water and our setup was modified for proper CO_2_ supply^58^.

### Fluorescence dissipation after photoactivation

Photoactivation was performed by briefly exposing a defined rectangular region of interest positioned across one side of the metaphase spindle to 405-nm laser. Live imaging was performed at 30 s intervals for 15 min. For fluorescence decay curves and half-life analyses, the measurements of fluorescence intensity decay for each blastomere were plotted against time and fitted into a double exponential curve *f*(*t*) = *A* × exp(−*k*_1_*t*) + *B* × exp(−*k*_2_*t*) using the cftool on MATLAB^43,59,60^. In this equation, *t* represents time; *A*, the less stable non-kMTs; *B*, the stable kMTs and *k*_1_ and *k*_2_ represent the decay rates of *A* and *B*, respectively. The half-life for each MT population was calculated as ln 2/*k*. Photobleaching was corrected for each measurement by imaging MII eggs exposed to 10 µM of the MT-stabilising agent Taxol, where MT turnover is minimal. Poleward flux velocity was calculated by determining the distance between the fluorescent mark on the spindle and the corresponding spindle pole at each time point^45,60^.

### Imaging analysis and statistics

All image processing and analysis was performed using ImageJ/Fiji^61^. No thresholding or masking was applied to the images and LUT brightness varies linearly. For centromere tracking in Fig. 4a, b, manual tracking was performed using the “Manual Tracking with TrackMate” feature of TrackMate plugin^62^ on Fiji. For metaphase plate width measurements in Fig. 4c, d, the distance between two lines drawn across each side of the metaphase plate was measured. The lines were drawn based on the MajSatTALE:mClover fluorescent signal and delimited the area occupied by all aligned chromosomes. Three dimensional reconstructions (Supplementary Fig. 5b; Supplementary Movies 1–3) and surface rendering (Supplementary Fig. 5b and Supplementary Movie 3) were generated using IMARIS 9.3. For MCAK fluorescence intensity analysis in Fig. 5f, g, background-subtracted fluorescence intensity values were obtained from a total of 100 kinetochores analysed from 10 embryos (10 kinetochores per embryo) for each group and statistical analysis was performed as an inter-embryo comparison using the average fluorescence intensity per embryo. All measurements analysed in this study were taken from distinct samples and samples were not measured repeatedly. Data were analysed using GraphPad Prism 7 software (GraphPad Software, La Jolla, CA, USA, www.graphpad.com). Shapiro–Wilk normality tests were applied where appropriate and either unpaired two-tailed *t* tests or two-tailed Mann–Whitney tests were applied. Statistical significance was considered when *P* < 0.05.

### Reporting summary

Further information on research design is available in the Nature Research Reporting Summary linked to this article.

## Supplementary information

Supplementary Information

Peer Review File

Description of Additional Supplementary Files

Supplementary Movie 1

Supplementary Movie 2

Supplementary Movie 3

Reporting Summary

Source Data

## Data Availability

The data related to the findings of this study are available within the manuscript and Supplementary Information, or from the corresponding author upon request. The source data underlying Figs. 1c, e, g, 2c, 3a, b, d, e, 4b, d, f, g, h, 5c, e, g, i, Supplementary Figs. 1b, d, 2a, b, e, 3b, 4a, b, 5c, d, 5e, f, h, 6a–d, 8a, b, d, e, g, h are provided as a Source Data file.
