# Supplementary material for: Tetraploidy causes chromosomal instability in acentriolar mouse embryos
Source: Nat Commun. 2019 Oct 23;10:4834. doi: 10.1038/s41467-019-12772-8 (PMC6811537; doi:10.1038/s41467-019-12772-8)
Supplement: Supplementary file 1 — Supplementary Information [file 41467_2019_12772_MOESM1_ESM.pdf]

Tetraploidy causes chromosomal instability in acentriolar mouse embryos

Paim and FitzHarris

Supplementary Figures

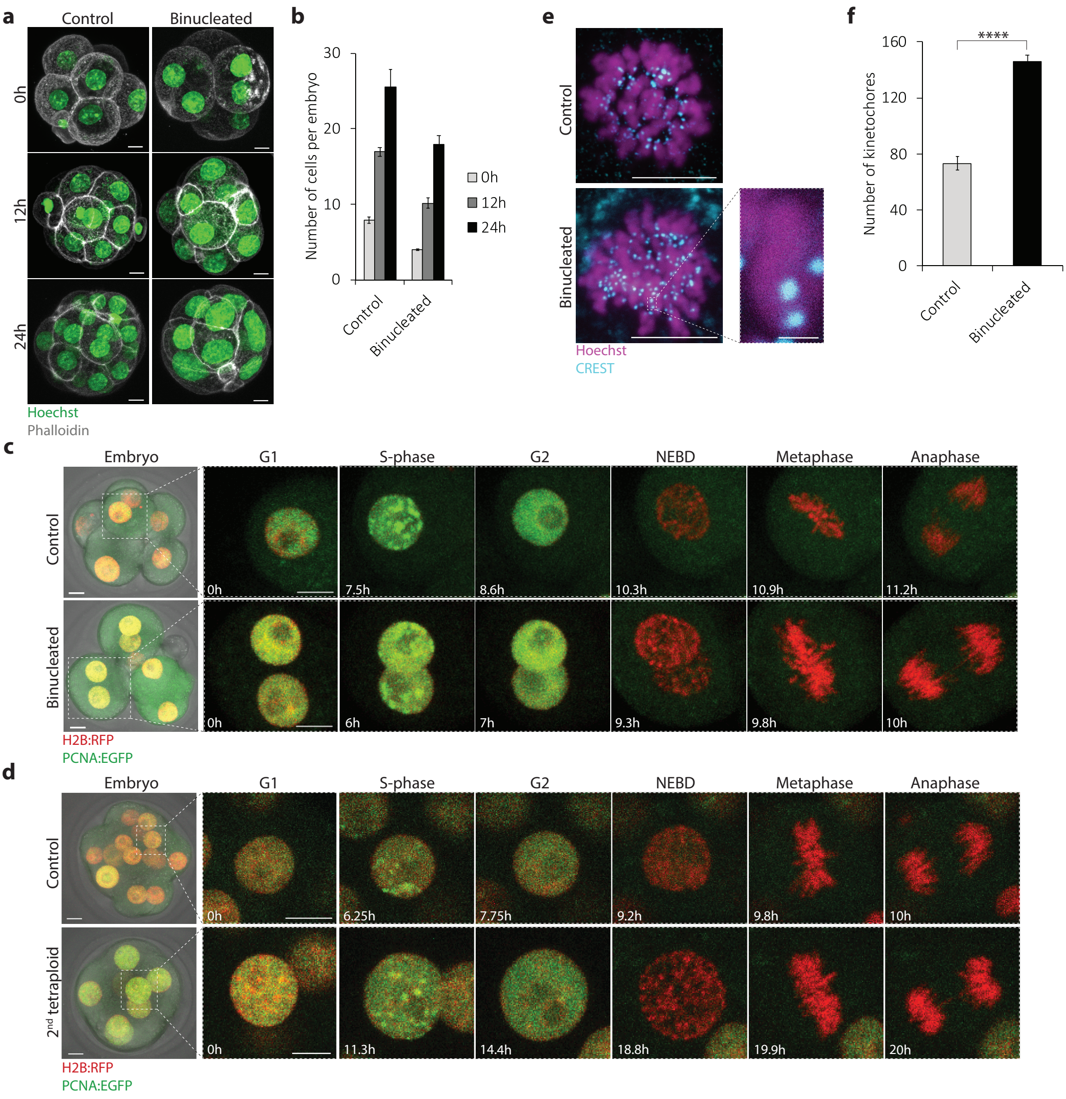

Supplementary Figure 1. The mouse embryo lacks a tetraploidy checkpoint. (a) Representative z-projections of embryos fixed 0h, 12h and 24h after exposure to either Latrunculin B (Binucleated) or DMSO (Control). **(b)** Quantification of number of cells per embryo 0h, 12h, and 24h after exposure to either DMSO (0h n=34 embryos; 12h n=31 embryos; 24h n=21 embryos) or Latrunculin B (0h n=27 embryos; 12h n=21 embryos; 24h n=23 embryos). **(c and d)** Representative z-projections of 8-cell control, 4-cell binucleated **(c)**, 16-cell control and 8-cell tetraploid embryos **(d)** expressing H2B:RFP (red) and PCNA:EGFP (green). Note that S-phase can be identified by the accumulation of PCNA foci at the nucleus demarking DNA replication. **(e)** Representative z-projections of metaphase-arrested controls and binucleated embryos. Note that treatment with Monastrol induces monopolar spindle formation, allowing for the better visualisation of individual kinetochores. **(f)** Quantification of number of kinetochores per cell in 8-cell control (n=5 blastomeres) and 4-cell binucleated embryos (n=8 blastomeres) *****P*<0.0001 (unpaired, two-tailed t test). Note that this approach allows for an approximation of kinetochore numbers (close to 80 kinetochores in controls and close to 160 kinetochores in tetraploids). Scale bars = 10µm, except for the chromosome zoom in (**e**), where scale bar = 1µm. Error bars represent SEM. NEBD=nuclear envelope breakdown.

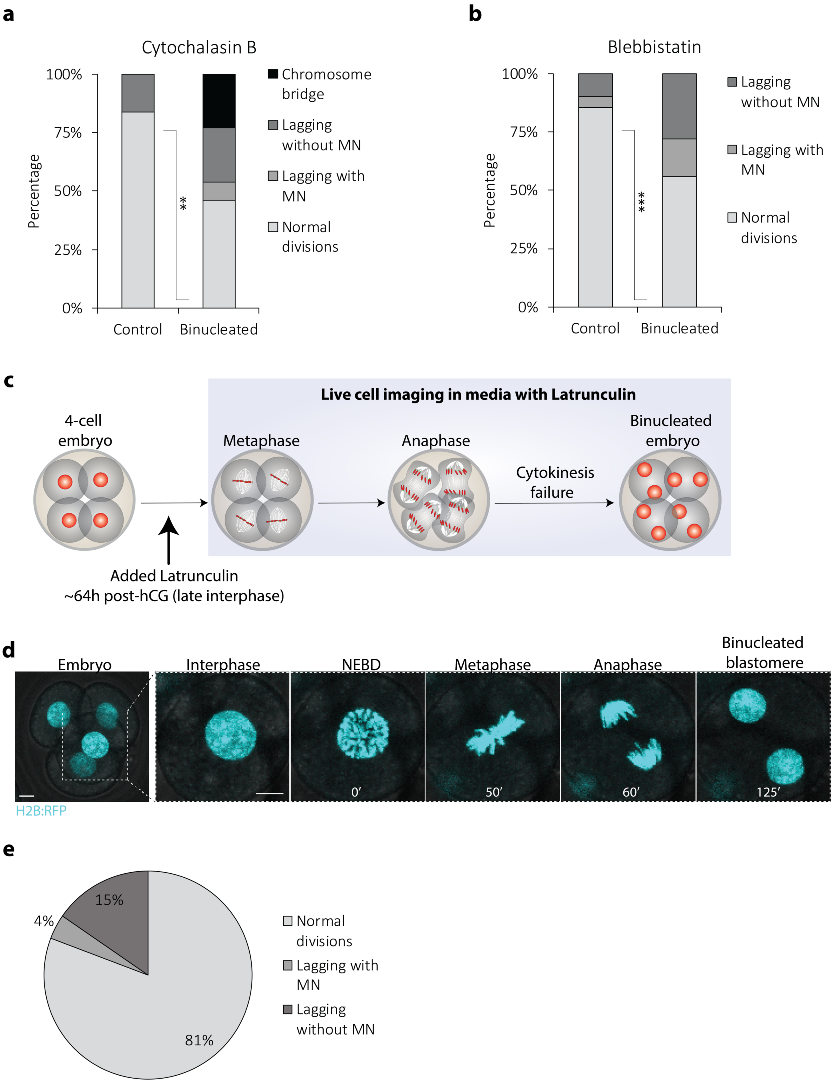

Supplementary Figure 2. The chromosome segregation errors observed after Latrunculin-induced binucleation are phenocopied by Cytochalasin B and Blebbistatin. (a and b) Percentage of chromosome segregation errors in 8-cell control and 4-cell binucleated embryos produced with either Cytochalasin B (control n= 62 divisions from 15 embryos; binucleated n=13 divisions from 7 embryos; ***P*=0.0072, unpaired, two-tailed Fisher's exact test) or Blebbistatin treatment (control n=90 divisions from 14 embryos; binucleated n=43 divisions from 16 embryos; ****P*=0.0004, unpaired, two-tailed Fisher's exact test). **(c)** Scheme illustrating the experimental design applied to assess the effects of actin depolymerization on chromosome segregation dynamics. 4-cell stage embryos at late interphase were live imaged in the presence of Latrunculin and the rate of chromosome segregation errors was assessed. **(d)** Representative z-projections of H2B:RFP-expressing 4-cell stage embryos live imaged in media containing Latrunculin B. **(e)** Percentage of chromosome segregation errors in 4-cell embryos imaged in media containing Latrunculin (n=26 divisions from 9 embryos). Chromosome segregation errors observed included: lagging chromosomes resulting in micronuclei formation (lagging with MN); lagging chromosomes that did not result in micronuclei formation (lagging without MN); and chromosome bridges. Scale bars = 10μm. NEBD=nuclear envelope breakdown.

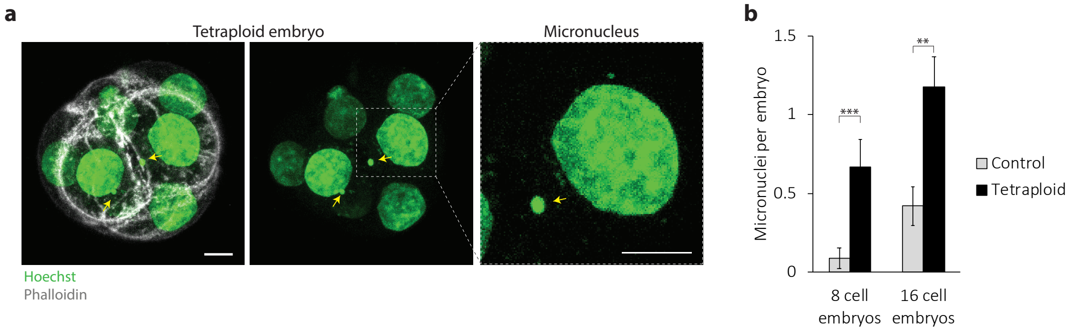

Supplementary Figure 3. Tetraploid embryos display high rates of micronuclei. (a) Representative z-projection of an 8-cell tetraploid embryo with two micronuclei (yellow arrows). **(b)** Rates of micronuclei per embryo in 8-cell controls (n=34 embryos), 8-cell tetraploid (n=21 embryos, ***P=0.0006, unpaired, two-tailed t test), 16-cell controls (n=31 embryos) and 16-cell tetraploid embryos (n=23 embryos, **P=0.0011, unpaired, two-tailed t test). Scale bars = 10µm. Error bars represent SEM.

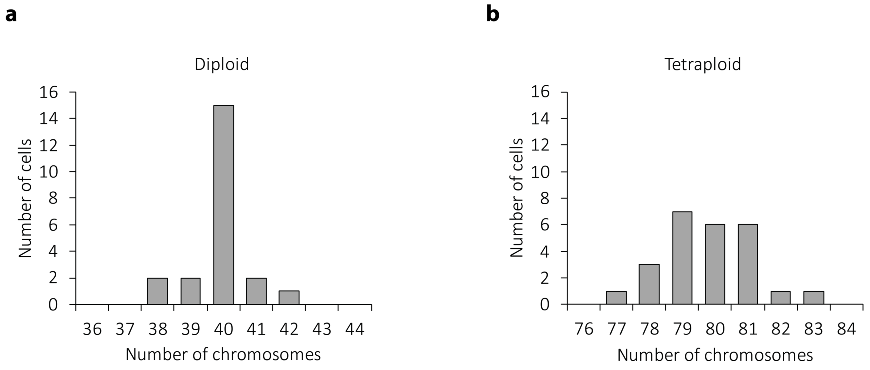

Supplementary Figure 4. Tetraploidy causes high levels of aneuploidy. (a) Histogram demonstrating the distribution of ploidy status in diploid blastomeres (n = 22 spreads). **(b)** Histogram demonstrating the distribution of ploidy status in tetraploid blastomeres (n= 25 spreads).

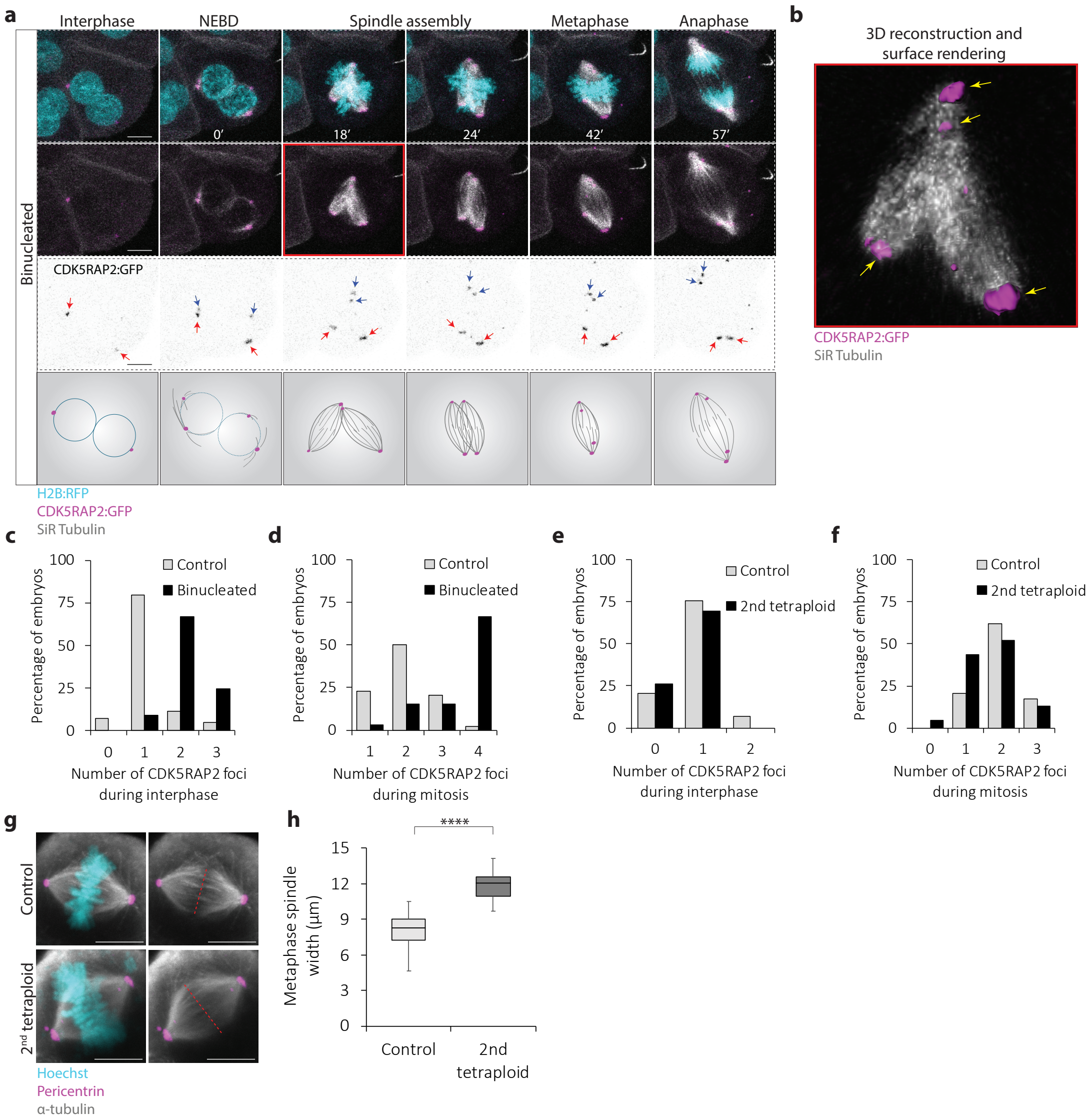

Supplementary Figure 5. Spindle assembly and MTOC dynamics in tetraploid embryos. (a) Representative z-projection and illustrations of a 4-cell binucleated embryo stained with SiR Tubulin (grey) and co-expressing H2B:RFP (cyan) and CDK5RAP2:GFP (magenta and inverted grey) chosen to illustrate an example of perpendicular spindle fusion. Two major microtubule organising centres (MTOCs) (red arrows) can be observed during interphase and two newly assembled MTOCs (blue arrows) can be observed during mitosis. **(b)** Three-dimensional reconstruction of the spindle fusion event in panel **(a)** (red square), with a surface rendering representation of spindle poles (magenta). Note the presence of four completely individualised spindle poles (yellow arrows), demarking the presence of two independent spindles during fusion. **(c and d)** Quantification of number of CDK5RAP2 foci during interphase **(c)** and mitosis **(d)** in 8-cell control (n=44 divisions from 11 embryos) and 4-cell binucleated embryos (n=33 divisions from 14 embryos). **(e and f)** Quantification of number of CDK5RAP2 foci during interphase **(e)** and mitosis **(f)** in 16-cell control (n=29 divisions from 9 embryos) and 8-cell tetraploid embryos (n=24 divisions from 9 embryos). **(g)** Representative immunofluorescence z-projections of a metaphase spindle of 16-cell control and 8-cell tetraploid embryo. **(h)** Quantification of metaphase spindle width in 16-cell controls (n= 31 spindles from 15 embryos) and 8-cell tetraploid embryos (n= 27 spindles from 14 embryos) ****P<0.0001 (unpaired, two-tailed t test). Scale bars = 10μm. Where box plots are shown, the centre line represents the median, the bounds of box represent the upper and lower quartiles and the whiskers represent minimum and maximum values. NEBD=nuclear envelope breakdown.

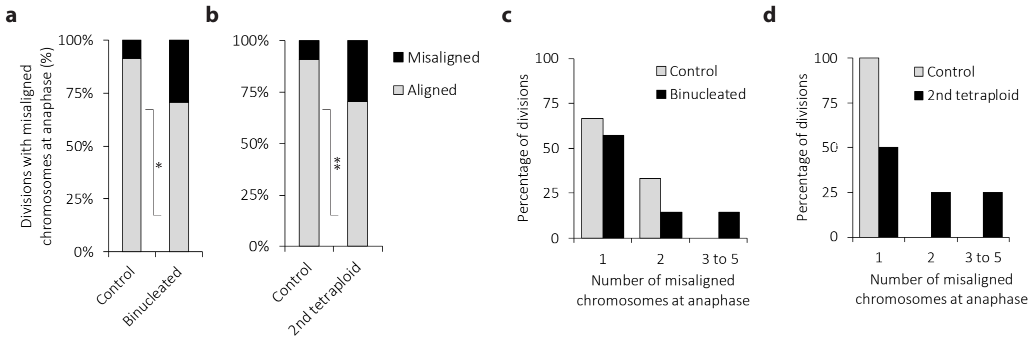

Supplementary Figure 6. High frequency of misaligned chromosomes in tetraploid embryos. (a and b) Percentage of divisions with severely misaligned chromosomes at anaphase onset in 8-cell controls (n= 47 divisions from 7 embryos), 4-cell binucleated embryos (n= 30 divisions from 8 embryos, *P=0.0412, two-tailed Fisher's exact test) **(a)**, 16-cell controls (n= 66 divisions from 7 embryos) and 8-cell tetraploid embryos (n= 50 divisions from 9 embryos, **P=0.0012, two-tailed Fisher's exact test) **(b)**. **(c and d)** Quantification of number of severely misaligned chromosomes visible at anaphase onset per cell division in 8-cell controls, 4-cell binucleated embryos **(c)**; 16-cell controls and 8-cell tetraploid embryos **(d)**.

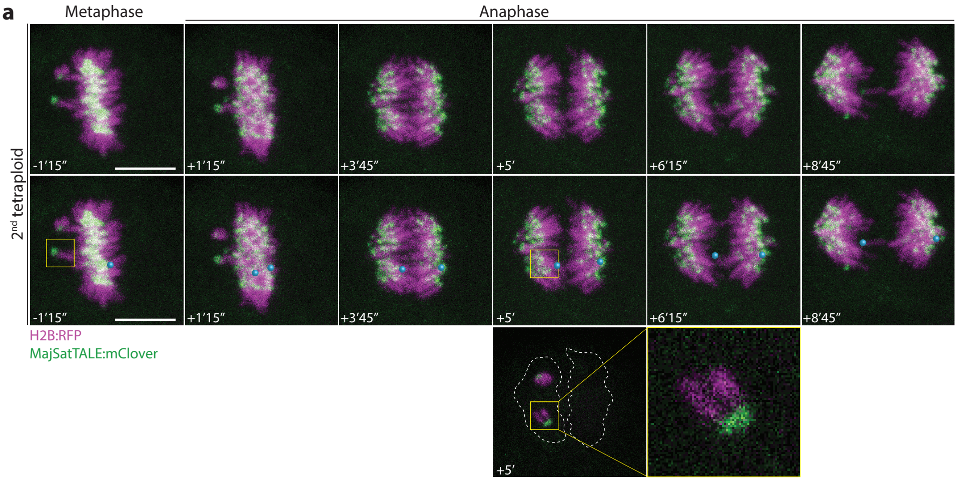

Supplementary Figure 7. Lagging chromosomes arise from fully aligned chromosomes. (a) A time-lapse image of mitosis in an 8-cell tetraploid embryo that exemplifies both anaphase chromosome lagging, and also misalignment at anaphase. Blue circles highlight a sister pair that was aligned at anaphase onset, but which gives rise to a lagging chromosome in anaphase. The yellow box indicates a sister pair that remains misaligned at the time of anaphase onset. The bottom row displays a reduced z-projection to clearly visualise the misaligned chromosome in mid anaphase. Note that both sister chromatids move towards the same spindle pole. Scale bars = 10μm.

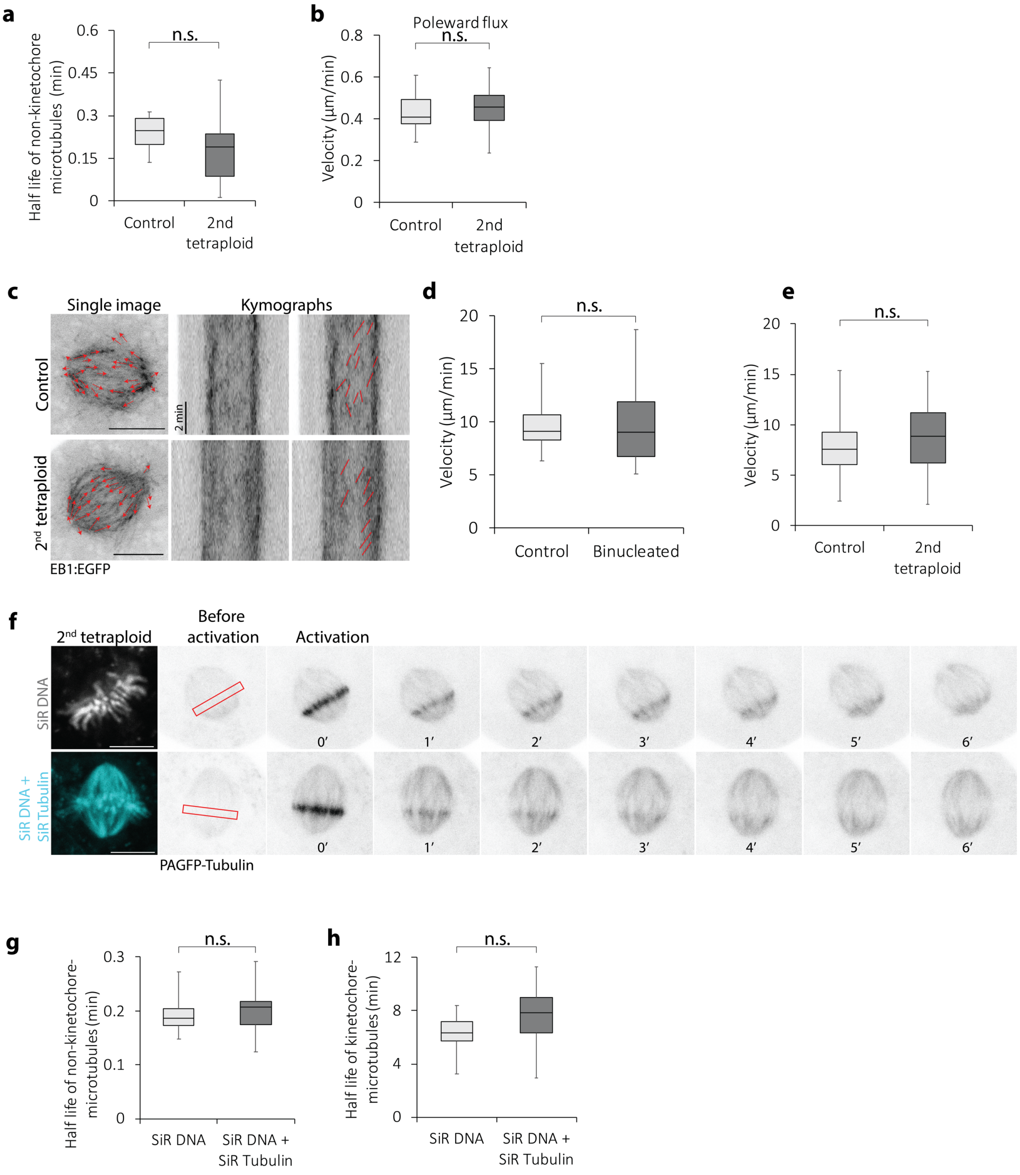

Supplementary Figure 8. Non-kinetochore-microtubule half-life, poleward flux and microtubule growth events are unchanged in tetraploid embryos. (a and b) Quantification of non-kinetochore-microtubule half-life in 16-cell control (n=11 blastomeres) and 8-cell tetraploid embryos (n=9 blastomeres) **(a)** and poleward flux in 16-cell control (n=11 blastomeres) and 8-cell tetraploid embryos (n=10 blastomeres) **(b)** (unpaired, two-tailed t test). **(c)** Representative z-projections and kymographs of control and tetraploid embryos expressing EB1:EGFP. **(d and e)** Quantification of microtubule growth velocity in 8-cell controls (n=36 tracks from 4 embryos), 4-cell binucleated (n=28 tracks from 3 embryos) (unpaired, two-tailed Mann-Whitney test) **(d)**, 16-cell controls (n=76 tracks from 8 embryos) and 8-cell tetraploid embryos (n=58 tracks from 6 embryos) (unpaired, two-tailed t test) **(e)**. **(f)** Representative time-lapse images of PAGFP-tubulin (inverted grey) in 8-cell tetraploid embryos labeled with either SiR DNA (grey) or SiR DNA + SiR Tubulin (cyan). **(g and h)** Quantification of non-kinetochore-microtubule half-life (unpaired, two-tailed Mann-Whitney test) **(g)** and kinetochore-microtubule half-life **(h)** in 8-cell tetraploid embryos labeled with either SiR DNA (n= 10 blastomeres) or SiR DNA + SiR Tubulin (n= 10 blastomeres) (unpaired, two-tailed Mann-Whitney test). Scale bars = 10µm. Where box plots are shown, the centre line represents the median, the bounds of box represent the upper and lower quartiles and the whiskers represent minimum and maximum values.

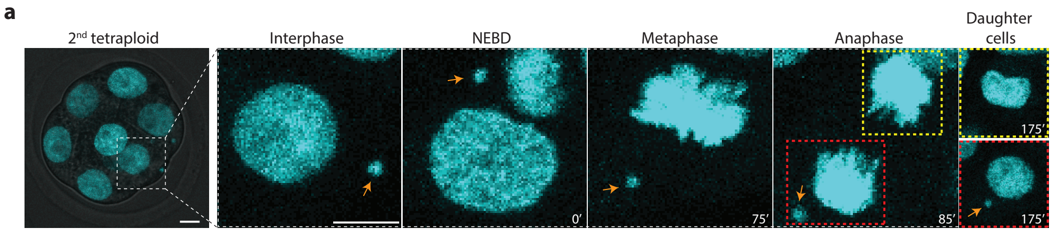

Supplementary Figure 9. Unilateral inheritance of micronuclei in tetraploid embryos. (a) Representative time-lapse images of mitosis in an 8-cell tetraploid embryo. A micronucleus originated from the previous cell division is observed (orange arrows). As anaphase takes place, the micronucleus is inherited by one of the daughter cells (red dashed square) without reincorporation in the principal nucleus, whereas the other daughter cell (yellow dashed square) remains without micronucleus. Note that the micronucleus remained visibly separate from the main chromosome mass throughout the process of cell division. The same unilateral inheritance pattern was observed in all 8 mitoses analysed. Scale bars = 10μm. NEBD=nuclear envelope breakdown.
