# Supplementary material for: Tetraploidy causes chromosomal instability in acentriolar mouse embryos
Source: Nat Commun. 2019 Oct 23;10:4834. doi: 10.1038/s41467-019-12772-8 (PMC6811537; doi:10.1038/s41467-019-12772-8)
Supplement: Supplementary file 3 — Description of Additional Supplementary Files [file 41467_2019_12772_MOESM3_ESM.pdf]

Description of Additional Supplementary Files

File name: Supplementary Movie 1
Description: Spindle assembly in an 8-cell diploid embryo. Three-dimensional reconstruction of mitosis in an 8-cell diploid embryo. Grey, microtubules; cyan, chromatin; magenta, spindle poles. Time is displayed in minutes.

File name: Supplementary Movie 2
Description: Spindle fusion in a 4-cell binucleated embryo. Three-dimensional reconstruction of mitosis in a 4-cell binucleated embryo. Grey, microtubules; cyan, chromatin; magenta, spindle poles. The movie demonstrates the formation of two individualised spindles that rapidly fuse and form a single bipolar spindle during the binucleated division. Time is displayed in minutes.

File name: Supplementary Movie 3
Description: Perpendicular spindle fusion in a 4-cell binucleated embryo. Three-dimensional reconstruction of mitosis in a 4-cell binucleated embryo. Grey, microtubules; magenta, spindle poles. The movie displays surface rendered spindle poles and a rotation of the image during spindle fusion to demonstrate that even in situations of perpendicular spindle fusion, two spindles are clearly distinguishable, without neighbouring poles connecting via microtubule bundles. Yellow arrows indicate spindle poles. Time is displayed in minutes.
