# Supplementary material for: Tetraploidy causes chromosomal instability in acentriolar mouse embryos
Source: Nat Commun. 2019 Oct 23;10:4834. doi: 10.1038/s41467-019-12772-8 (PMC6811537; doi:10.1038/s41467-019-12772-8)
Supplement: Supplementary file 7 — Reporting Summary [file 41467_2019_12772_MOESM7_ESM.pdf]

nature research

Corresponding author(s): Greg FitzHarris

Last updated by author(s): Sep 5, 2019

Reporting Summary

Nature Research wishes to improve the reproducibility of the work that we publish. This form provides structure for consistency and transparency in reporting. For further information on Nature Research policies, seeAuthors & Referees and theEditorial Policy Checklist .

Statistics

For all statistical analyses, confirm that the following items are present in the figure legend, table legend, main text, or Methods section.

n/a	Confirmed	
☐	☒	The exact sample size (*n*) for each experimental group/condition, given as a discrete number and unit of measurement
☐	☒	A statement on whether measurements were taken from distinct samples or whether the same sample was measured repeatedly
☐	☒	The statistical test(s) used AND whether they are one- or two-sided<br>*Only common tests should be described solely by name; describe more complex techniques in the Methods section.*
☒	☐	A description of all covariates tested
☐	☒	A description of any assumptions or corrections, such as tests of normality and adjustment for multiple comparisons
☐	☒	A full description of the statistical parameters including central tendency (e.g. means) or other basic estimates (e.g. regression coefficient) AND variation (e.g. standard deviation) or associated estimates of uncertainty (e.g. confidence intervals)
☐	☒	For null hypothesis testing, the test statistic (e.g. *F*, *t*, *r*) with confidence intervals, effect sizes, degrees of freedom and *P* value noted<br>*Give P values as exact values whenever suitable.*
☒	☐	For Bayesian analysis, information on the choice of priors and Markov chain Monte Carlo settings
☒	☐	For hierarchical and complex designs, identification of the appropriate level for tests and full reporting of outcomes
☒	☐	Estimates of effect sizes (e.g. Cohen's *d*, Pearson's *r*), indicating how they were calculated

Our web collection on statistics for biologists contains articles on many of the points above.

Software and code

Policy information about availability of computer code

Data collection	All images were collected using either a Leica SP8, SP5 confocal microscope including a Leica Application Suite Advanced Fluorescence software (LAS AF version 2.7.3.9723) or a Leica Application Suite X (LAS X version 2.02.15022) or a Zeiss Axio observer, equipped with an Axiocam and Apotome including a ZEN software.
Data analysis	All image processing and analysis was performed with Fiji/Image J version 1.52g or 2.0 (open source image processing software - https://imagej.net/Fiji)<br>All statistical analyses were performed using GraphPad Prism 7 software (GraphPad Software, La Jolla California USA, www.graphpad.com)<br>Microtubule half-life in Figures 5c, S8a-b, S8g-h was calculated based on fluorescence intensity decay plots obtained using MATLAB R2017b (9.3.0.713579) https://www.mathworks.com/products/matlab.html<br>Three dimensional reconstructions and surface rendering presented in Figure S5b and Supplementary Movie 1, 2 and 3 were generated using IMARIS 9.3 software (https://imaris.oxinst.com/)

For manuscripts utilizing custom algorithms or software that are central to the research but not yet described in published literature, software must be made available to editors/reviewers. We strongly encourage code deposition in a community repository (e.g. GitHub). See the Nature Research guidelines for submitting code & software for further information.

Data

Policy information about availability of data

All manuscripts must include a data availability statement. This statement should provide the following information, where applicable:

- Accession codes, unique identifiers, or web links for publicly available datasets
- A list of figures that have associated raw data
- A description of any restrictions on data availability

The data related to the findings of this study are available within the manuscript and Supplementary Information, or from the corresponding author upon request.

The source data underlying Figs 1c, 1e, 1g, 2c, 3a, 3b, 3d, 3e, 4b, 4d, 4f, 4g, 4h, 5c, 5e, 5g, 5i, Supplementary Figures 1b, 1d, 2a, 2b, 2e, 3b, 4a, 4b, 5c, 5d, 5e, 5f, 5h, 6a, 6b, 6c, 6d, 8a, 8b, 8d, 8e, 8g, 8h are provided as a Source Data file.

Field-specific reporting

Please select the one below that is the best fit for your research. If you are not sure, read the appropriate sections before making your selection.

[x] Life sciences [ ] Behavioural & social sciences [ ] Ecological, evolutionary & environmental sciences

For a reference copy of the document with all sections, see nature.com/documents/nr-reporting-summary-flat.pdf

Life sciences study design

All studies must disclose on these points even when the disclosure is negative.

Sample size	Sample sizes are based on nearly 20 years experience of doing this type of investigation in this system.
Data exclusions	No data was excluded
Replication	All experiments were performed on a minimum of 2 experimental days, with similar results.
Randomization	Embryos from 2 or more mice were pooled and therefore randomised before experimental intervention.
Blinding	Blinding not possible. All measurements are objective rather than subjective.

Reporting for specific materials, systems and methods

We require information from authors about some types of materials, experimental systems and methods used in many studies. Here, indicate whether each material, system or method listed is relevant to your study. If you are not sure if a list item applies to your research, read the appropriate section before selecting a response.

Materials & experimental systems

n/a	Involved in the study	
[ ]	[x]	Antibodies
[x]	[ ]	Eukaryotic cell lines
[x]	[ ]	Palaeontology
[ ]	[x]	Animals and other organisms
[x]	[ ]	Human research participants
[x]	[ ]	Clinical data

Methods

n/a	Involved in the study	
[x]	[ ]	ChIP-seq
[x]	[ ]	Flow cytometry
[x]	[ ]	MRI-based neuroimaging

Antibodies

Antibodies used

Primary antibodies:
CREST human antisera (gift from Marvin J Fritzler);
beta-tubulin anti-mouse (supplier: Sigma Aldrich; catalog #:T4026; lot #; 043M4785; clone: TUB2.1);
MCAK anti-rabbit (gift from Duane Compton);
MAD2 anti-rabbit (supplier: BioLegend; catalog #: 924601; lot #: B205130; clone: Poly1946).
Pericentrin anti-mouse (BD Biosciences; catalog #611814; Lot # 3277704)
alpha-tubulin anti-rabbit (Abcam; catalog #AB18251; Lot # GR265350-1)

Secondary antibodies:
Alexa 633 anti-human (supplier: Life Technologies/ThermoFisher; catalog #: A21091; lot #: 1613055)
Alexa 546 anti-human (supplier: Invitrogen/ThermoFisher; catalog #: A21089; lot #: 1800815)
Alexa 488 anti-human (supplier: Life Technologies/ThermoFisher; catalog #: A11013; lot #: 1668688)
Alexa 488 anti-rabbit (supplier: Life Technologies/ThermoFisher; catalog #: A11008 ; lot #: 1735088)
Alexa 633 anti-mouse (supplier: Life Technologies/ThermoFisher; catalog #: A21050; lot #: 1744778)

Conjugated antibodies:
Alexa Fluor 555 Phalloidin (supplier: Invitrogen/ThermoFisher; catalog #: A34055; lot #: 1615008).

Validation

CREST human antisera has been previously applied for immunofluorescence in peer-reviewed publications by our team (Intrinsically Defective Microtubule Dynamics Contribute to Age-Related Chromosome Segregation Errors in Mouse Oocyte Meiosis-I. Nakagawa and FitzHarris. Current Biology 7(3), 1040-1047 (2017)).

beta-tubulin anti-mouse has been previously validated for immunofluorescence as evidenced on the supplier's website (https://www.sigmaaldrich.com/catalog/product/sigma/t4026?lang=en&region=CA) and by previous peer-reviewed publications (Vascular-targeting activity of ZD6126, a novel tubulin-binding agent. Micheletti G, et.al. Cancer Research 63(7), 1534-1537, (2003))

MCAK anti-rabbit has been previously applied for immunofluorescence in peer-reviewed publications by Dr. Duane Compton (who provided the antibody) (Analysis of mitotic microtubule-associated proteins using mass spectrometry identifies astrin, a spindle-associated protein. Mack and Compton. PNAS 98(25), 14434-9 (2001)) and by our team (MCAK regulates chromosome alignment but is not necessary for preventing aneuploidy in mouse oocyte meiosis I. Illingworth et al. Development 137(13) 2133–2138 (2010).

MAD2 anti-rabbit has been previously validated for immunofluorescence as evidenced on the supplier's website (https://www.biolegend.com/ja-jp/products/anti-mad2-antibody-11080) and by previous peer-reviewed publications by our team (Cell-Size-Independent Spindle Checkpoint Failure Underlies Chromosome Segregation Error in Mouse Embryos. Vazquez-Diez, C et al. Current Biology 2019 https://doi.org/10.1016/j.cub.2018.12.042).

Pericentrin anti-mouse has been previously validated for immunofluorescence as evidenced on the supplier's website (http://www.bdbiosciences.com/us/reagents/research/antibodies-buffers/cell-biology-reagents/cell-biology-antibodies/purified-mouse-anti-mouse-pericentrin-30pericentrin/p/611814).

alpha-tubulin anti-rabbit has been previously validated for immunofluorescence as evidenced on the supplier's website (https://www.abcam.com/alpha-tubulin-antibody-ab18251.html#top-488).

Animals and other organisms

Policy information about studies involving animals; ARRIVE guidelines recommended for reporting animal research

Laboratory animals

Species: Mus musculus
Strain: Crl:CD1(ICR) - Charles River Laboratories
Sex: Female
Age: 2-3 months

Species: Mus musculus
Strain: Crl:CD1(ICR) - Charles River Laboratories
Sex: Male
Age: 4-12 months

Wild animals

This study did not involve wild animals.

Field-collected samples

This study did not involve samples collected from the field.

Ethics oversight

All experiments were approved by the Centre de Recherche du Centre Hospitaliaire de l'Universite de Montreal (CRCHUM) Comite Institutionnel de Protections des Animaux (CIPA). Protocol number: IP18034GFs

Note that full information on the approval of the study protocol must also be provided in the manuscript.
