# Supplementary material for: Tetraploidy causes chromosomal instability in acentriolar mouse embryos
Source: Nat Commun. 2019 Oct 23;10:4834. doi: 10.1038/s41467-019-12772-8 (PMC6811537; doi:10.1038/s41467-019-12772-8)
Supplement: Supplementary file 2 — Peer Review File [file 41467_2019_12772_MOESM2_ESM.pdf]

Editorial Note: Parts of this Peer Review File have been redacted as indicated to maintain the confidentiality of unpublished data.

Reviewers' comments:

Reviewer #1 (Remarks to the Author):

In this manuscript, Gomes Paim and FitzHarris examine the consequences of inducing tetraploidy on aneuploidy in the preimplantation mouse embryo. Excess of centrosomes is shown to promote aneuploidy in other cell types. Therefore the authors use their approach to dissect the role of tetraploidy independently of centrosome number.

They find that tetraploid embryos display substantial aneuploidy and therefore conclude that there are other mechanisms, independent of centrosomes, leading to aneuploidy and chromosome instability. Their experimental approach and findings of high aneuploidy in tetraploid embryos are very interesting. However, the mechanism explaining these observations need to be studied in much more detail.

The proposal at the end of the paper is that the excess of chromosomes and kinetochores dilute a microtubule destabilizing factor (the atypical kinesin MCAK), normally required to fix errors and prevent aneuploidy. But they only observe reduced fluorescence intensity of MCAK in the tetrapoloid condition. The authors should support their conclusions by manipulating the levels of this regulatory protein (for example by overexpression, knockdown approaches, mutated forms, etc) to aggravate or rescue aneuploidy. They should also try to increase / decrease aneuploidy levels in control mouse embryos by manipulating MCAK.

These experiments would strengthen their conclusion that '…doubled kinetochore numbers in tetraploid blastomeres presents an overburden on the ability to recruit microtubule-destabilising factors, such as MCAK'.

Additional points:

- Fig. 4f shows MCAK specks at kinetochores, which are fainter in tetraploid than control cells. However, since tetraploid cells carry double the number of kinetochores, wouldn't we expect to see twice the amount of specs (this is clear in Fig. S1c for CREST for example but not in Fig. 4f)?

- All stats, n numbers, etc should be in the figures, not in the main text. Currently the main text is constantly interrupted by numbers, which can be shown in the figures.

- Fig. 4g is critical for this paper. N = 10 cells is a rather low number. Can the authors increase their sampling for this? Maybe improve the statistical values?

- In line 191, the authors refer to S4 instead of S6.

- What does 'MN' stands for in Fig 1c (i.e. lagging with or without MN)?

- 'Weakening the SAC' is a vague statement, the authors be more specific here. What do they mean by a weaker SAC?

Reviewer #2 (Remarks to the Author):

In this manuscript, Paim and FitzHarris chemically induce cytokinesis failure and tetraploidization at the 4 to 8 cell transition of mouse embryos. These embryos continue to divide, and exhibit an

elevated rate of lagging and bridge chromosomes in the next two divisions. Timelapse microscopy revealed that, rather than passing through a multipolar intermediate, most binucleate cells formed two separate spindles that fused to permit bipolar segregation of DNA. Mitotic duration was elongated in tetraploid cells, which recruited Mad2 to unattached kinetochores, consistent with a functional spindle assembly checkpoint. Photoactivatable GFP-tubulin was used to show an increased half-life of kinetochore microtubules in cells undergoing the second tetraploid division, which also recruit less of the microtubule depolymerase MCAK. The authors conclude that tetraploidy causes chromosomal instability (CIN) by altering microtubule dynamics without inducing multipolar spindles.

A novel mechanism for tetraploidy to induce CIN would be of broad interest. Though the authors may have discovered such a mechanism, the evidence as presented here is currently insufficient to substantiate the major claims of the manuscript, namely that multipolar spindles are not the cause of CIN and that microtubule dynamics are altered by tetraploidy.

The first major conclusion of the manuscript is that tetrapolar blastomeres assemble bipolar spindles without first establishing multipolar spindles. The evidence for this is in figure 2b, 2e, and S4a. Of these three examples, the spindle in S4a does appear to first establish a multipolar (tripolar) spindle before forming a bipolar spindle. This would be consistent with the mechanism previously reported for tetraploidy to cause CIN (Ganem et al, Nature 2009; Silkworth et al, PLoS One 2009). Movies showing spindle formation as well as quantitation of the frequency with which bipolar spindles formed de novo, versus from fusion of separate spindles or fusion of multipolar spindles would help to establish this claim. Also, it is necessary to report how frequently cells were imaged during mitosis to establish that multipolar intermediates would have been visualized if present.

The experiment with photoactivatable GFP-tubulin in figure 4a-c is used to establish the second major claim of the manuscript, that tetraploidy increases the stability of kinetochore microtubules, which leads to lagging chromosomes and CIN. However, these experiments are performed in the presence of SiR-tubulin, which consists of the SiR fluorophore conjugated to the microtubule stabilizing taxane docetaxel. Microtubules act as a sink for taxane binding, and the taxanes concentrate substantially intracellularly due to microtubule binding (Jordan et al, PNAS 1993; Yvon et al, Mol Biol Cell 1999). Since tetraploid spindles contain ~2x more microtubules than diploid spindles, they would be expected to contain ~2x docetaxel as well. It is unclear that the ~2x increase in microtubule halflife in the tetraploid spindles is due to tetraploidy rather than an increase in the relative concentration of docetaxel.

A movie showing robust Mad2 recruitment shortly after NEB followed by loss would help to support that claim that the spindle checkpoint is not further weakened by tetraploidy in embryos, as would quantification of total Mad2 signal intensity, not just the number of positive kinetochores. Figure S5a and b indicate that tetraploid embryos enter anaphase with substantially more misaligned chromosomes than euploid embryos (~25% versus ~7%). Since the tetraploid embryos also spend more time in mitosis, what is the proposed mechanism for this if the spindle checkpoint is unaffected?

Since tetraploid cells would be expected to have ~2x the concentration of MCAK, why would MCAK recruitment be impaired in tetraploid cells? Is Aurora B (which also destabilizes improper kinetochore microtubules) similarly affected?

It would be helpful to supplement figure 1g with an additional graph showing the range and frequency of chromosome numbers in formerly tetraploid cells.

The statement that “establishment of correct (amphitelic) chromosome attachment prior to anaphase occurs by correction of previously mis-attached kinetochores” (lines 178-179) assumes that all kinetochores initially form inappropriate attachments, which is not always the case.

There are numerous potential causes of chromosomal instability (CIN) in cancer. While the specific causes of CIN at work in primary and metastatic tumors remain elusive, it is unlikely that there is a single cause. The conclusion that “tetraploidy…is responsible for the high levels of CIN in cancer” (lines 54-55) is not well supported and should be amended, as should the similar statement on lines 228-229.

The control division in figure 2d has a lagging chromosome.

Line 147: Fig. 2C-D should be Fig. 2D-E.

Line 191 refers to Supplementary Figure 6, not Supplementary Figure 4.

The lower case a, b, and c’s in figure 3a-b are not defined.

Reviewer #3 (Remarks to the Author):

Tetraploidy causes chromosomal instability in acentriolar mouse embryos

Paim et al. study how early mouse embryos, which are naturally acentriolar, adjust to a cytokinesis failure-induced tetraploidization event during the next mitosis. They surprisingly find that, unlike in somatic cells where extra centrioles lead to multipolar spindles and merotely associated with a high chromosome segregation error rate, tetraploid mouse embryos do not form multipolar spindles but nevertheless display numerous chromosome segregation errors during anaphase. By analyzing microtubule dynamics in tetraploid blastomeres, the authors conclude that altered kinetochore-microtubule dynamics that lead to defects in chromosome attachments are the cause of the chromosome segregation errors. They thus propose that tetraploidisation can drive chromosomal instability (CIN) by a novel mechanism that does not involve a multipolar spindle intermediate, but instead is induced by altered kinetochore microtubule interactions. As the authors observed that the kinetochore level of the depolymerizing kinesin-13 MCAK is reduced in tetraploid blastomeres, they propose a model of tetraploidy-induced CIN that relies on the limited amount of
microtubule-destabilising factors (such as MCAK) when the kinetochore number is doubled in tetraploid cells. Insufficient amounts of kinetochore-loaded MCAK would prevent individual kinetochores from maintaining sufficient microtubule turnover, leading to merotelic attachments, chromosome segregation errors and ultimately CIN.
Overall, the study is of high quality and the main conclusions well supported by impressive live imaging on a difficult model system (i.e. the early mouse embryo). On the other end, the proposed model for tetraploidisation-induced CIN is very speculative at this stage as the study is mainly descriptive and lacks significant functional validation of the model.

Major comments:
1-An important analysis, which is currently missing, is the direct assessment of kinetochore behavior by live imaging in control vs tetraploid blastomeres. This could be performed by following and tracking GFP-CenpC in live to determine if the overall kinetochore behavior is perturbed as is predicted by the authors.

2-Could the authors try a direct validation of their model for tetraploidization-induced CIN, by over-expressing MACK in tetraploid embryos and testing if this rescues (even partially) the high error rate in chromosome segregation?

3-A better description of the method used (timing of drug addition/removal) for inducing cytokinesis failure should be added to the main figures (perhaps on Fig. 1a). The authors should

also explain how they performed what appears to be a very important control experiment (continuous drug treatment) and, which is currently presented as a single graph on Fig. S2d.

4-How do the authors explain the discrepancy between the low levels of merotelic/unattached kinetochores observed upon cold treatment (Fig. 4d and e) and the high level of lagging chromosomes in tetraploid blastomeres (Fig. 1d and e)?

Minor comments:
1-Line 133: “Upon contact, the spindles fused by either sliding together or rotating towards each other depending on the initial angle of contact until they eventually became a single bipolar spindle (Fig. 2b and c; Supplementary Fig. 4a), as observed in Xenopus extract spindles in close appositionand also mouse zygotes29,30.”
The very first observation of two bipolar spindles fusing together into a bigger structure was in fact obtained in Fmn2-/- mouse oocytes after MI polar body extrusion failure and was reported in Dumont et al, Dev. Biol., 2007. This work should be cited here.

2-Scale bars must be included on every image panel.

3-Panel 1f is not cited in the manuscript.

4-Image panels on Figure 2 should be bigger.

5-Panels S1c and d are first cited in the text after panels S1e and f.

6-The spindles displayed on Fig. 4a have completely different shapes. Could the authors comment on this? Is the difference due to the doubled number of chromosomes?

Reviewer #1 (Remarks to the Author):
In this manuscript, Gomes Paim and FitzHarris examine the consequences of inducing tetraploidy on aneuploidy in the preimplantation mouse embryo. Excess of centrosomes is shown to promote aneuploidy in other cell types. Therefore, the authors use their approach to dissect the role of tetraploidy independently of centrosome number.

They find that tetraploid embryos display substantial aneuploidy and therefore conclude that there are other mechanisms, independent of centrosomes, leading to aneuploidy and chromosome instability. Their experimental approach and findings of high aneuploidy in tetraploid embryos are very interesting. However, the mechanism explaining these observations need to be studied in much more detail.

The proposal at the end of the paper is that the excess of chromosomes and kinetochores dilute a microtubule destabilizing factor (the atypical kinesin MCAK), normally required to fix errors and prevent aneuploidy. But they only observe reduced fluorescence intensity of MCAK in the tetrapoloid condition. The authors should support their conclusions by manipulating the levels of this regulatory protein (for example by overexpression, knockdown approaches, mutated forms, etc) to aggravate or rescue aneuploidy. They should also try to increase / decrease aneuploidy levels in control mouse embryos by manipulating MCAK.

These experiments would strengthen their conclusion that '…doubled kinetochore numbers in tetraploid blastomeres presents an overburden on the ability to recruit microtubule destabilising factors, such as MCAK'.

We thank the reviewer for positive and helpful comments.

We fully agree that manipulating MCAK levels would vastly strengthen the link between reduced kinetochore MCAK and segregation error in tetraploids, a point also made by Reviewer#3. We are delighted to say that we have now been able to add this analysis.

We overexpressed either MCAK:GFP or GFP alone in the second tetraploid division. Whereas GFP was homogeneously distributed, MCAK:GFP decorated kinetochores, as expected. Consistent with previous results, GFP-only controls exhibited many lagging chromosomes in the second tetraploid division. Strikingly, however, the number of lagging chromosomes and other defects was substantially and significantly reduced in MCAK:GFP tetraploids. Thus MCAK is under-repesented at kinetochores, and introducing ectopic MCAK:GFP can reduce the likelihood of errors. These results are consistent with chromosomally unstable cancer cell lines, where MCAK (and other kinesin 13s) are under-represented, and MCAK over expression can 'rescue' chromosome segregation errors (Bakhoum et al., 2009 Nat Cell Biol). Our data thus support the assertion that the kinetochore MCAK reduction is a likely reason for the reduced MT turnover and increased segregation error and CIN in tetraploid embryos.

We thank the reviewer again, as we feel this experiment strengthens the paper. <u>Please see Figure 5h and i, and lines 262-268.</u>

Additional points:
Fig. 4f shows MCAK specks at kinetochores, which are fainter in tetraploid than control cells. However, since tetraploid cells carry double the number of kinetochores, wouldn't we expect to see twice the amount of specs (this is clear in Fig. S1c for CREST for example but not in Fig. 4f)?

Yes there are twice as many kinetochores, but this is not always obvious in single images of metaphase aligned chromosomes, where the kinetochores are often close together. The fact that kinetochore number is doubled is made clearly in Supplementary Figure 1e, where monastrol was used to disrupt the spindle and therefore disperse the chromosomes for easier viewing.

All stats, n numbers, etc should be in the figures, not in the main text. Currently the main text is constantly interrupted by numbers, which can be shown in the figures.

This has now been changed as suggested, with the exception of a small number of occasions where a value is essential to understand the result (<u>see line 80 for example</u>).

Fig. 4g is critical for this paper. N = 10 cells is a rather low number. Can the authors increase their sampling for this? Maybe improve the statistical values?

Many thanks for this comment, which has made us realise that we had explained this experiment poorly, and in the process under-represented the quality of the data. 10 kinetochores were scored in each cell, for a <u>total n of 100 in each group</u>. This value is consistent with (or greater than) similar analyses by leading groups in our field (see for example Yoshida et al, Dev Cell, 2015, Yun et al Cell Cycle, 2014). We stated significance in terms of inter-embryo variation, for a P-value of 0.02. In fact other studies simply compared all kinetochores, which would have given $P<0.00001$ in our case.

Therefore, in response to the reviewers comment, we have changed the presentation of the data. Kinetochores are now individually represented in a scatter plot, but statistical significance is still stated in terms of inter-embryo variance as this is the more conservative approach, and this is explained clearly in the figure legend (See Figure 5g). We have also far more carefully explained the experimental design and the approach to data analysis, as described above (see Materials and Methods, lines 411-415 and Figure legend, lines 706-708). We hope that these changes help the reader to better appreciate the soundness of the data.

In line 191, the authors refer to S4 instead of S6.

Many thanks. This has been fixed.

What does 'MN' stands for in Fig 1c (i.e. lagging with or without MN)?

'MN' stands for micronuclei. This has now been clarified in the figure legend.

'Weakening the SAC' is a vague statement, the authors be more specific here. What do they mean by a weaker SAC?

With hindsight we agree we explained this poorly. In most cells a misaligned chromosome/kinetochore typically recruits SAC proteins which leads to enforcement of the spindle checkpoint, and prevention of anaphase onset. Our previous work (Vazquez-Diez et al, Curr Biol 2019) showed that, in early embryos, misaligned chromosomes/kinetochores do indeed recruit SAC proteins, but then fail to enforce the arrest, which we term a 'weak SAC'. The reviewer is right that since this is a recent observation and not a well-known phenomenon, we should have described it much better. We have now done so at lines 169-171. See also further discussion on the embryo SAC weakness below in response to Reviewer#2.

Reviewer #2 (Remarks to the Author):

In this manuscript, Paim and FitzHarris chemically induce cytokinesis failure and tetraploidization at the 4 to 8 cell transition of mouse embryos. These embryos continue to divide, and exhibit an elevated rate of lagging and bridge chromosomes in the next two divisions. Timelapse microscopy revealed that, rather than passing through a multipolar intermediate, most binucleate cells formed two separate spindles that fused to permit bipolar segregation of DNA. Mitotic duration was elongated in tetraploid cells, which recruited Mad2 to unattached kinetochores, consistent with a functional spindle assembly checkpoint.

Photoactivatable GFPtubulin was used to show an increased halflife of kinetochore microtubules in cells undergoing the second tetraploid division, which also recruit less of the microtubule depolymerase MCAK. The authors conclude that tetraploidy causes chromosomal instability (CIN) by altering microtubule dynamics without inducing multipolar spindles.

A novel mechanism for tetraploidy to induce CIN would be of broad interest. Though the authors may have discovered such a mechanism, the evidence as presented here is currently insufficient to substantiate the major claims of the manuscript, namely that multipolar spindles are not the cause of CIN and that microtubule dynamics are altered by tetraploidy.

The first major conclusion of the manuscript is that tetrapolar blastomeres assemble bipolar spindles without first establishing multipolar spindles. The evidence for this is in figure 2b, 2e, and S4a. Of these three examples, the spindle in S4a does appear to first establish a multipolar (tripolar) spindle before forming a bipolar spindle. This would be consistent with the mechanism previously reported for tetraploidy to cause CIN (Ganem et al, Nature 2009; Silkworth et al, PLoS One 2009). Movies showing spindle formation as well as quantitation of the frequency with which bipolar spindles formed de novo, versus from fusion of separate spindles or fusion of multipolar spindles would help to establish this claim. Also, it is necessary to report how frequently cells were imaged during mitosis to establish that multipolar intermediates would have been visualized if present.

We thank the reviewer for their comments, and are pleased to hear they find it interesting and of broad interest. We understand that the reviewer seeks re-assurance that the accumulation of merotelic attachments in embryos is not attributable to multipolar spindles, as it is in somatic cells with supernumery centrioles.

Our data is clear that in the second tetraploid division (where there is only one nucleus), spindle formation is normal, without multipolarity. Similarly in the first tetraploid division, where two spindles form and fuse, in many cases such as the one shown in Figure 2 there is no suggestion of multipolarity as the spindles fuse end-on and slide together. Thus the vast majority of tetraploid divisions display no hint of a multipolar division. However, the reviewer is

understandably focusing on the example shown in Supplementary Figure 5, in which the spindles fuse in a more perpendicular manner, and that wonders whether this may in fact constitute a multipolar spindle.

Note that we chose the example in Supplementary Figure 5 to show one of the clearest examples of perpendicular fusion to illustrate the difference with end-on fusion. However, even in this extreme case, the scenario is quite distinct to multipolar spindes as described in the literature for at least two reasons. **Firstly,** and most importantly, multipolar spindles in the literature (including in our previous work in oocytes, Nakagawa and FitzHarris, Curr Biol 2017) exhibit pronounced MT bundles between each adjacent pole, giving the spindle a 'triangular' (or quadratic etc) appearance. Note that even in the example we showed here that is not the case, and the two spindles are clearly distinguishable throughout fusion, without neighbouring poles connecting via MT bundles. This is mechanistically important, as it is the communication between multiple poles that is essential for merotelic attachment in somatic (cancer) cells – but this is not what happens in embryos. **Secondly,** in somatic cells multipolar spindles persist for a substantial period of time, allowing merotelic attachments to form (Yang et al, 2008; Ganem et al 2009). This is not the case in embryos, where the spindles fuse rapidly without pause, the whole process taking only a few minutes. Thus, in the vast majority of tetraploid blastomeres there is no suggestion of multipolarity, and even the minority scenario of perpendicular spindle fusion in the binucleated division is critically different to multipolarity as described in somatic cells.

We realise now that we should have taken more time explaining and justifying this. Thus as a result of the reviewers comment we have now made the following changes and additions.

1. We have added further sections to the text to explain more clearly, as we have above, how even the minority situation of perpendicular spindle fusion is distinct from multipolar spindles described in somatic cells (see lines 148-150).
2. As suggested by the reviewer, we have included movies of spindle fusion (see Supplementary Movies 2 and 3), which makes clear that even in the case of perpendicular spindle fusion, true multipolarity is not seen.
3. We have included a 3D reconstruction in Supplementary Figure 5 to further illustrate that the poles are not in communication.
4. We have represented MTs and poles separately in Supplementary Figure 5a to make spindle structure clearer.
5. We have described the imaging parameters in more detail to underline the point that a multipolar phase has not simply been missed by slow imaging (see 146-147).

The experiment with photoactivatable GFPtubulin in figure 4ac is used to establish the second major claim of the manuscript, that tetraploidy increases the stability of kinetochore microtubules, which leads to lagging chromosomes and CIN. However, these experiments are performed in the presence of SiRtubulin, which consists of the SiR fluorophore conjugated to the microtubule stabilizing taxane docetaxel. Microtubules act as a sink for taxane binding, and the taxanes concentrate substantially intracellularly due to microtubule binding (Jordan et al, PNAS 1993; Yvon et al, Mol Biol Cell 1999).

Since tetraploid spindles contain ~2x more microtubules than diploid spindles, they would be expected to contain ~2x docetaxel as well.
It is unclear that the ~2x increase in microtubule halflife in the tetraploid spindles is due to tetraploidy rather than an increase in the relative concentration of docetaxel.

The reviewer is right that SiR Tubulin is a taxol derivative, and we had not formally excluded the possibility of 'side effects' of the label upon MT dynamics. To directly address this concern we have performed an additional control experiment, comparing MT turnover using PAGFP-tubulin in tetraploid embryos either in the presence or absence of SiR Tubulin. Tellingly, there was no difference in turnover rate, either of kinetochore MTs or non-kinetochore MTs. We are grateful to the reviewer for suggesting this, and are happy to have ruled this out. This new data is presented as Supplementary Figure 8f-h, and is described in the results in 241-245.

We suspect that the lack of this control underpinned the reviewers hesitation that the change in MT dynamics was fully supported by the data, and hope this addition allays their concerns.

A movie showing robust Mad2 recruitment shortly after NEB followed by loss would help to support that claim that the spindle checkpoint is not further weakened by tetraploidy in embryos, as would quantification of total Mad2 signal intensity, not just the number of positive kinetochores.

We thank the reviewer for the suggestion. We have now performed and included live imaging experiments examining a GFP fusion protein of MAD1 (MAD1:2GFP), a second kinetochore SAC protein with similar dynamics to MAD2, the use of which we have already optimized. MAD1:2EGFP is recruited to kinetochores right after NEBD, followed by gradual and complete loss of signal in both the first and second tetraploid divisions, as is the case in the MAD2 immunofluorescence experiment. This new data is presented in Figure 3f and g and described at lines 180-183.

Figure S5a and b indicate that tetraploid embryos enter anaphase with substantially more misaligned chromosomes than euploid embryos (~25% versus ~7%). Since the tetraploid embryos also spend more time in mitosis, what is the proposed mechanism for this if the spindle checkpoint is unaffected?

Thank you for this comment. Indeed, our data did not previously explain *why* there should be more misaligned chromosomes at anaphase onset in tetraploid blastomeres. In response to this comment, and also the request from Reviewer #3 to perform centromere/kinetochore tracking, we have now added a major dataset using 4D tracking to analyse chromosome movement during chromosome alignment (Figure 4), which explains the phenomenon.

We labelled centromeres using MajSat-TALE-mClover, and used 4D tracking analysis to appraise centromere movements in detail. The data shows no difference in centromere velocities between 2N and 4N cells either in metaphase or anaphase. Moreover, the tightness of the metaphase plate is unaffected. Interestingly, however, our data shows that even in 2N embryos, aligned chromosomes occasionally become displaced, 'wandering' off the metaphase plate transiently before becoming aligned again. Strikingly whilst the distance moved away from the metaphase plate for a given sister-pair is similar for 2N and 4N cells, the duration of these events is prolonged in 4N cells. Importantly, almost all of the chromosomes in 4N cells that are seen to be misaligned at anaphase onset are chromosomes that were previously aligned, have subsequently wandered off the metaphase plate, and have not returned to the metaphase plate in time for anaphase.

The fact that anaphase is not inhibited by the misaligned chromosomes is consistent with our previous work that the SAC is insensitive to misalignments in embryos. And, as explained in Figure 3, there is no evidence that the SAC is further weakened in tetraploids. Rather, our data show that subtly altered chromosome behavior causes displacement events to become prolonged, increasing the probability that anaphase will occur when there is a misaligned sister pair. In addition, this new dataset also allows us to assert more clearly that, similar to somatic cells (Thompson & Compton, JCB 2008; Cimini et al., JCB 2001), lagging anaphase chromosomes are generally correctly aligned prior to anaphase.

This data appears as an entirely new Figure 4, plus a new Supplementary Figure 7 and is described at lines 190-221. We are happy that reviewer 2 and 3 have asked for this, as this additional data vastly increases our understanding of the defects in tetraploid embryos.

Since tetraploid cells would be expected to have ~2x the concentration of MCAK, why would MCAK recruitment be impaired in tetraploid cells?

Indeed this is an excellent question and we don't really know. The answer could involve a failure of signaling required for MCAK to be recruited (see Andrews et al., Dev Cell 2004; Lan et al., Curr Biol 2004 for example), or dosage compensation associated with tetraploidy (see Stingele et al., Mol Sys Biol 2012 for example). Attempting to work this out is a future goal, for which we plan to collaborate with the Petropoulos lab (Montreal), expert in omics approaches in embryos. In the meantime we realise the discussion on this was a little weak, and we have now strengthened this. Please see lines 286-291.

Is Aurora B (which also destabilizes improper kinetochore microtubules) similarly affected?

Yes this is an interesting question. Addressing this is a little more complicated in mouse oocytes and embryos than in somatic cells, as they express a third Aurora Kinase, called AuroraC. The functions and localisations of the three AurKs (A,B,C) are overlapping, complicated, and yet to be fully worked out (see Schindler et al PNAS 2012, Ngyuen et al Curr Biol 2018). Therefore, as a result of the reviewers comment, we set out address this using a pan-AurK antibody recently employed in the Nguyen 2018 study. Despite extensive optimization and alternative approaches, the quality of the immunofuorescence images we obtained, whilst good enough to comment on Aurora localisation, are not suitable for quantitative analysis looking for moderate changes in protein localisation. Note that the same is true for isoform specific antibodies. Below is one of the better examples of the pan-AurK antibody, along with an MCAK image for comparison. Note that whereas MCAK antibodies yield images appropriate for quantitative analysis, the signal with pan-AurK antibodies is not robust enough. It is a shame we have not been able to add this, but would rather not add measurements in which we do not have full confidence. Note that, as explained above, our discussion of this question in the text has improved in the meantime. Please see lines 283-291.

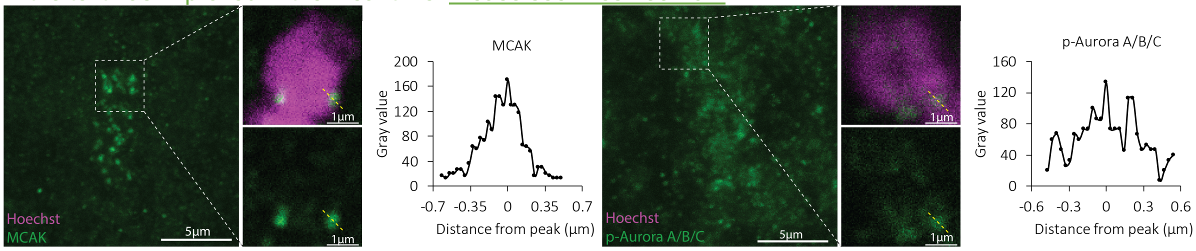

It would be helpful to supplement figure 1g with an additional graph showing the range and frequency of chromosome numbers in formerly tetraploid cells.

Thank you for this suggestion. We have now added this as a new Supplementary Figure 4 and have included in lines 110 and 112.

The statement that "establishment of correct (amphitelic) chromosome attachment prior to anaphase occurs by correction of previously misattached kinetochores" (lines 178 179) assumes that all kinetochores initially form inappropriate attachments, which is not always the case.

Thank you for your comment. We have now replaced this sentence in lines 225-226.

There are numerous potential causes of chromosomal instability (CIN) in cancer. While the specific causes of CIN at work in primary and metastatic tumors remain elusive, it is unlikely that there is a single cause. The conclusion that "tetraploidy…is responsible for the high levels of CIN in cancer" (lines 54 55) is not well supported and should be amended, as should the similar statement on lines 228 229.

Thank you. Yes these sentences were a little over-enthusiastic. They have now been tempered. Please see lines 52-54 and lines 292-293.

The control division in figure 2d has a lagging chromosome.

Many thanks - we have now replaced the control division in Fig 2d with a more representative example.

Line 147: Fig. 2CD should be Fig. 2DE.

Thank you. This has been fixed.

Line 191 refers to Supplementary Figure 6, not Supplementary Figure 4.

Many thanks - this has now been fixed.

The lower case a, b, and c's in figure 3ab are not defined.

Many thanks. This has now been added in the figure legend.

Reviewer #3 (Remarks to the Author):

Tetraploidy causes chromosomal instability in acentriolar mouse embryos Paim et al. study how early mouse embryos, which are naturally acentriolar, adjust to a cytokinesis failure induced tetraploidization event during the next mitosis. They surprisingly find that, unlike in somatic cells where extra centrioles lead to multipolar spindles and merotely associated with a high chromosome segregation error rate, tetraploid mouse embryos do not form multipolar spindles but nevertheless display numerous chromosome segregation errors during anaphase. By analyzing microtubule dynamics in tetraploid blastomeres, the authors conclude that altered kinetochore microtubule dynamics that lead to defects in chromosome attachments are the cause of the chromosome segregation errors.

They thus propose that tetraploidisation can drive chromosomal instability (CIN) by a novel mechanism that does not involve a multipolar spindle intermediate, but instead is induced by altered kinetochore microtubule interactions. As the authors observed that the kinetochore level of the depolymerizing kinesin13 MCAK is reduced in tetraploid blastomeres, they propose a model of tetraploidy induced CIN that relies on the limited amount of microtubule destabilizing factors (such as MCAK) when the kinetochore number is doubled in tetraploid cells. Insufficient amounts of kinetochore loaded MCAK would prevent individual kinetochores from maintaining sufficient microtubule turnover, leading to merotelic attachments, chromosome segregation errors and ultimately CIN. Overall, the study is of high quality and the main conclusions well supported by impressive live imaging on a difficult model system (i.e. the early mouse embryo). On the other end, the proposed model for tetraploidization induced CIN is very speculative at this stage as the study is mainly descriptive and lacks significant functional validation of the model.

Major comments:

1 – An important analysis, which is currently missing, is the direct assessment of kinetochore behavior by live imaging in control vs tetraploid blastomeres. This could be performed by following and tracking GFPCenpC in live to determine if the overall kinetochore behavior is perturbed as is predicted by the authors.

Kineochore tracking, such as has been presented in oocytes and somatic cells with GFP-tagged kinetochore proteins, has not previously been presented in embryos. We can tell the reviewer that we have been trying this for some time, and kinetochore probes that work well in oocytes are more problematic in early embryos. We have however optimized the use of the centromeric probe MajSat-TALE:mClover, which allows beautiful 4D tracking of centromere pairs, and have now presented this data in the new Figure 4.

As described above in response to Reviewer #2, the data shows no difference in centromere movement velocities between 2N and 4N cells either in metaphase or anaphase. Moreover, overall chromosome alignment as measured by the tightness of the metaphase plate is unaffected. Interestingly, however, our data shows that even in 2N embryos, aligned chromosomes occasionally become displaced, 'wandering' off the metaphase plate transiently before becoming aligned again. Strikingly whilst the distance moved away from the metaphase plate for a given sister-pair is similar for 2N and 4N cells, the duration of these events is prolonged in 4N cells. Strikingly, almost all of the chromosomes in 4N cells that are seen to be misaligned at anaphase onset are chromosomes that were previously aligned, have subsequently wandered off the metaphase plate, and have not returned to the metaphase plate in time for anaphase. Thus, subtly altered chromosome behavior causes displacement events to become prolonged, increasing the probability that anaphase will occur when there is a misaligned sister pair. The dataset also allows us to assert more clearly that, similar to somatic cells, lagging anaphase chromosomes are generally correctly aligned prior to anaphase. The addition of this data greatly strengthens the paper, and we are thus very greatful for the suggestion.

This data appears as the new Figure 4, plus a new Supplementary Figure 7and is described at 190-221.

2 Could the authors try a direct validation of their model for tetraploidization induced CIN, by overexpressing MACK in tetraploid embryos and testing if this rescues (even partially) the high error rate in chromosome segregation?

As discussed in detail in response to reviewer#1 above, this is an excellent suggestion, and we are very happy that we have been able to add this data as the new Figure 5h and i. The data shows that, consistent with work from cancer cells, introducing ectopic MCAK-GFP reduces the incidence of segregation defect.

We thank the reviewer for this suggestion, as we feel adding this experiment has strengthened the paper.

3 A better description of the method used (timing of drug addition/removal) for inducing cytokinesis failure should be added to the main figures (perhaps on Fig. 1a). The authors should also explain how they performed what appears to be a very important control experiment (continuous drug treatment) and, which is currently presented as a single graph on Fig. S2d.

Thank you. We have now added more details regarding the timing of of drug addition and removal in the scheme presented in Figure 1a. We have also included a new illustration in Supplementary Figure 2c describing the experimental design for continuous Latrunculin exposure, and have described this in more detail in the manuscript (see Materials and Methods, lines 324-329).

4 How do the authors explain the discrepancy between the low levels of merotelic/unattached kinetochores observed upon cold treatment (Fig. 4d and e) and the high level of lagging chromosomes in tetraploid blastomeres (Fig. 1d and e)?

Yes we understand the confusion here. Whereas it is normal to report anaphase defects in terms of % of cells with a defect, MT attachments (merotelic, unattached etc) are commonly reported in terms of % kinetochores with a given attachment type. Since theoretically only one kinetochore needs to be misattached at the onset of anaphase to cause a lagging chromosome, relatively low proportions of misattached kinetochores (on a kinetochore-by-kinetochore basis) potentially give rise to very high proportions of cells having at least one defect – which is indeed the case in our data.

As a result of the comment we have considered other ways of presenting the data. However, other approaches present different complications to the reader, and the way we have done it is standard in studies of this type (Kitajima et al., 2011 Cell; Mogessie & Schuh, 2017 Science; Nakagawa & FitzHarris, 2017 Curr Bio; Shomper et al., 2014 Cell Cycle). Therefore we have left the figures as they were, but have added extra clarification at lines 248-254 to explain more carefully that the increase in misattachments we observe in tetraploid cells is very consistent with the increase in segregation errors we see. We thank the reviewer for the comment and hope that the additional text makes this easier to follow for the reader.

Minor comments:

1 Line 133: "Upon contact, the spindles fused by either sliding together or rotating towards each other depending on the initial angle of contact until they eventually became a single bipolar spindle (Fig. 2b and c; Supplementary Fig. 4a), as observed in Xenopus extract spindles in close appositionand also mouse zygotes29,30." The very first observation of two bipolar spindles fusing together into a bigger structure was in fact obtained in Fmn2/ mouse oocytes after MI polar body extrusion failure and was reported in Dumont et al, Dev. Biol., 2007. This work should be cited here.

Thank you. This reference has now been included in lines 140-141.

2 Scale bars must be included on every image panel.

The scale bars are now included in every image panel.

3 Panel 1f is not cited in the manuscript.

This has now been fixed in the manuscript. Please see lines 109 and 112.

4 Image panels on Figure 2 should be bigger.

We have now re-arranged Figure 2 in order to increase the size of the panels.

5 Panels S1c and d are first cited in the text after panels S1e and f.

Thank you for spotting that. This has now been fixed in the figure and the text. Please see lines 85 and 87.

6 The spindles displayed on Fig. 4a have completely different shapes. Could the authors comment on this? Is the difference due to the doubled number of chromosomes?

Yes the reviewer is correct. The diameter of the metaphase plate is greater in tetraploids, leading to a broader spindle. The reviewer is right that we had not mentioned this in the paper. We have now added a new dataset in Supplementary Figure 5g and h and added in lines 155-158 to make this point clearly.

Reviewers' comments:

Reviewer #1 (Remarks to the Author):

The authors have satisfactorily addressed all my initial points and questions.
They also included very important functional experiments (manipulating MCAK levels) to strengthen their paper, so I can fully support publication of this study.

Reviewer #2 (Remarks to the Author):

The revised manuscript from Paim and FitzHarris is improved over the original submission. The response to the reviewers concerns was well considered and the experimental extensions worthwhile. Several issues remain.

It is not clear that lagging chromosomes caused by altered microtubule turnover represent the main cause of chromosome missegregation in tetraploid divisions. The evidence for the argument that “of the lagging chromosomes observed, none of them originated from a misaligned chromosome” comes from figure S7. However, this does not appear to provide sufficient resolution to discriminate which centromere becomes lagging.

The defect that seems most common is chromosomes that are misaligned at the start and remain misaligned. The number of chromosomes that fail to align is higher in tetraploid divisions (figure S6), consistent with a weakened SAC. These misaligned chromosomes may provide the most substantial contribution to the observed aneuploidy. Although some Mad1 and Mad2 are recruited to unattached kinetochores, this is not sufficient to demonstrate that the SAC is not weakened.

The type of defect described in figure 4e-h, in which an aligned chromosome becomes misaligned prior to anaphase onset, doesn’t result in a lagging or bridge chromosome. It doesn’t seem like this type of defect was quantified in figure 1c or e, but it could provide a substantial contribution to chromosome segregation, assuming both sisters remain together. Do both sister chromatids move together or is this an example of cohesin fatigue where the sister chromatids come apart (and are likely to be segregated accurately)? A movie would help to demonstrate this.

Along these lines, is overexpression of MCAK:GFP sufficient to reduce aneuploidy? If it is, this would support that argument that lagging chromosomes produced by altered microtubule dynamics are the main source of chromosome missegregation.

If lagging with MN is ≥25% in figure 1c and 1e, why are MN only ~1% in fixed cells in figure S3?

Figure S5h says the metaphase plate is larger in the 2nd tetraploid division, but figure 4c-d says it isn’t. Which is correct?

Chromosomes that are aligned, become misaligned and then realign in control cells are graphed in figure 4f, but not depicted in figure 4e (or S7). The example shows unaligned chromosomes aligning. A representative image sequence is necessary to show that aligned chromosomes misalign and realign in control cells.

The cartoon under the center panel in figure 5d does not seem to accurately represent the image.

In figure S6c and d, does “3≤5” mean 3 to 5 (ie 3-5)?

The red circles described in the legend in figure S7 are not visible in the figure.

If the distinct lower-case letters represent statistical significance between groups at $P<0.05$, but

the specific P values are not listed, what is the benefit of using distinct symbols rather just use a single symbol (normally *) to indicate P<0.05?

Reviewer #3 (Remarks to the Author):

The authors have added a significant amount of new data in the revised version of the manuscript. They have satisfactorily addressed all my questions and concerns. I am therefore convinced about their main conclusions. This manuscript should thus be published without further delay.

Reviewers' comments:

Reviewer #1 (Remarks to the Author):

The authors have satisfactorily addressed all my initial points and questions.
They also included very important functional experiments (manipulating MCAK levels) to strengthen their paper, so I can fully support publication of this study.

Many thanks again for the thoughtful comments in the first round of reviews.

Reviewer #3 (Remarks to the Author):

The authors have added a significant amount of new data in the revised version of the manuscript. They have satisfactorily addressed all my questions and concerns. I am therefore convinced about their main conclusions. This manuscript should thus be published without further delay.

Many thanks again for the helpful suggestions.

Reviewer #2 (Remarks to the Author):

The revised manuscript from Paim and FitzHarris is improved over the original submission. The response to the reviewers concerns was well considered and the experimental extensions worthwhile. Several issues remain.

Many thanks. We are happy to have allayed the major concerns raised at first review.

It is not clear that lagging chromosomes caused by altered microtubule turnover represent the main cause of chromosome missegregation in tetraploid divisions. The evidence for the argument that "of the lagging chromosomes observed, none of them originated from a misaligned chromosome" comes from figure S7. However, this does not appear to provide sufficient resolution to discriminate which centromere becomes lagging.

Yes, we are grateful for this observation, which along with the related comments below helped us realise that we had not done enough to explain the difference between lagging and misalignment. To clear this up we have added two extra panels of supplemental data that are described and discussed in a new paragraph found at lines 293-310.

First, as requested, we present a more detailed example of centromere tracking that shows clearly that at least in some cases of extreme misalignment we can see that the misaligned sister pair is clearly inherited in its entirety by one daughter cell (Supplementary Figure 7). Therefore, misalignments can indeed cause aneuploidy as the reviewer had imagined. **Second**, we add further data indicating that lagging chromosomes that lead to micronuclei formation must cause aneuploidy. Our previous paper (Vazquez-Diez et al., 2016, PNAS) shows that, in contrast to somatic cells, in embryos the micronucleus almost never re-joins the main nucleus, but is instead repeatedly 'inherited' by daughter cells. This necessarily drives aneuploidy, and is almost certainly the explanation for some otherwise hard-to-explain specific patterns of aneuploidy seen in human IVF clinics. We have now added the imaging data showing that the same 'MN inheritance' pattern occurs in tetraploid embryos also (New Supplementary Figure 9).

Thus, the two defects are mechanistically separate and, at least in tetraploid mouse embryos, both can cause aneuploidy. Please see the new paragraph at lines 293-310, and the new panels found in Supplementary Figure 7 and Supplementary Figure 9. Many thanks again for the helpful comments.

The defect that seems most common is chromosomes that are misaligned at the start and remain misaligned. The number of chromosomes that fail to align is higher in tetraploid divisions (figure S6), consistent with a weakened SAC. These misaligned chromosomes may provide the most substantial

contribution to the observed aneuploidy. Although some Mad1 and Mad2 are recruited to unattached kinetochores, this is not sufficient to demonstrate that the SAC is not weakened.

In fact, the most common defect observed in our tracking data is chromosomes that align and subsequently misalign prior to anaphase onset rather than an overall failure in chromosome alignment (Figure 4).

Our data show that kinetochore SAC component dynamics are apparently normal (now shown both in live and fixed experiments), and though there are more misaligned chromosomes at the moment of anaphase onset, this is not a result of a shortening of mitosis, rather mitosis is indeed extended somewhat consistent with an active SAC. We therefore conclude that it is the altered chromosome alignment dynamics (Fig4), rather than a wholesale SAC defect, that is responsible for the misalignment at anaphase onset.

That said, we do take the reviewers point that these experiments do not definitively prove that there is zero impact upon SAC signalling. We have therefore better clarified this and softened the language around this point. Please see lines 187-190.

The type of defect described in figure 4e-h, in which an aligned chromosome becomes misaligned prior to anaphase onset, doesn't result in a lagging or bridge chromosome. It doesn't seem like this type of defect was quantified in figure 1c or e, but it could provide a substantial contribution to chromosome segregation, assuming both sisters remain together. Do both sister chromatids move together or is this an example of cohesin fatigue where the sister chromatids come apart (and are likely to be segregated accurately)? A movie would help to demonstrate this.

Please see above. [redacted]

Along these lines, is overexpression of MCAK:GFP sufficient to reduce aneuploidy? If it is, this would support that argument that lagging chromosomes produced by altered microtubule dynamics are the main source of chromosome missegregation.

We assume this specific request is now less pertinent given that we have better explained the contribution of laggings and misalignments. We'd also add that the reviewer may not have realised quite what an undertaking this specific experiment would be. Chromosome spread experiments such as the one we present in Figure1 are extremely rare in embryos as they are extremely difficult and time consuming. Just that data panel alone in Fig1f-g took several months to compile. Note that the rescue of lagging by MCAK is not 100%, and more than double the amount of spreads would be needed to achieve statistical power. The extra information we would get beyond what we already know, on what is a minor point in the paper, would not justify the amount of work and time necessary to do this.

If lagging with MN is ≥25% in figure 1c and 1e, why are MN only ~1% in fixed cells in figure S3?

This is a misunderstanding: in Supplementary Figure 3 it is 1 cell per embryo that had a micronucleus (not 1%). This equates to ~10% of cells. The reason it is not the full 25% is because there are almost never any errors in the first few several mitoses (see Vazquez-Diez et al, PNAS, 2016).

Figure S5h says the metaphase plate is larger in the 2nd tetraploid division, but figure 4c-d says it isn't. Which is correct?

Again, this is a misunderstanding. The measurement shown in Figure 4c-d is of chromosome congression. The data in Supplementary Figure 5h shows the width of the metaphase spindle, as requested by reviewer #3. To make this clearer we have relabelled the chart in Supplementary Figure 5h as 'metaphase spindle width'.

Chromosomes that are aligned, become misaligned and then realign in control cells are

graphed in figure 4f, but not depicted in figure 4e (or S7). The example shows unaligned chromosomes aligning. A representative image sequence is necessary to show that aligned chromosomes misalign and realign in control cells.

This was already shown in Figure 4e, and highlighted as orange and green circles. We have now added a sentence to the figure legend to draw more attention to it. Please see lines 700-701 and 704-705.

The cartoon under the center panel in figure 5d does not seem to accurately represent the image.

Many thanks. We have improved this cartoon and added some further explanation to the figure legend. Please see lines 721-724.

In figure S6c and d, does “3≤5” mean 3 to 5 (ie 3-5)?

Many thanks. We have now corrected the x axis label on Supplementary Figure 6c and d.

The red circles described in the legend in figure S7 are not visible in the figure.

Many thanks for spotting this. The circles were actually blue. We have now corrected the figure legend in Supplementary Figure 7.

If the distinct lower-case letters represent statistical significance between groups at $P<0.05$, but the specific P values are not listed, what is the benefit of using distinct symbols rather just use a single symbol (normally *) to indicate $P<0.05$?

Many thanks. We have reverted to asterisks as suggested and have included the P values in the figure legend. Please see lines 667 and 669.

REVIEWERS' COMMENTS:

Reviewer #2 (Remarks to the Author):

The authors have satisfactorily addressed my questions and concerns with one minor exception - based on the available evidence, it would be more appropriate for “affected” to be replaced by “abrogated” in the subheading “Spindle Assembly Checkpoint activity is not affected in tetraploid embryos”.

Reviewers' comments:

Reviewer #2 (Remarks to the Author):

The authors have satisfactorily addressed my questions and concerns with one minor exception - based on the available evidence, it would be more appropriate for “affected” to be replaced by “abrogated” in the subheading “Spindle Assembly Checkpoint activity is not affected in tetraploid embryos”.

Many thanks, we are happy to have allayed the concerns raised in the previous round of revisions.
We have now replaced the word “affected” by “abrogated” at lines 167 and 664.
